# Association of volatile organic compound exposure with metabolic syndrome and its components: a nationwide cross-sectional study

**DOI:** 10.1186/s12889-024-18198-2

**Published:** 2024-03-02

**Authors:** Rui Dong, Dongchun Chang, Chao Shen, Ya Shen, Zhengkai Shen, Ting Tian, Jie Wang

**Affiliations:** 1 Department of Fundamental and Community Nursing, School of Nursing, Nanjing Medical University, Nanjing, China; 2 Nanjing Municipal Center for Disease Control and Prevention, Nanjing, China; 3 Department of Integrated Service and Management, Jiangsu Province Center for Disease Control and Prevention, Nanjing, China; 4 Jiangsu Provincial Center for Disease Control and Prevention, Institute of Nutrition and Food Safety, Nanjing, China

**Keywords:** Volatile organic compounds, Central obesity, Triglyceride, High-density lipoprotein, National health and nutrition examination survey, Metabolism

## Abstract

**Background:**

Metabolic syndrome (MetS) is a health issue consisting of multiple metabolic abnormalities. The impact of exposure to volatile organic compounds (VOCs) on MetS and its components remains uncertain. This study aimed to assess the associations of individual urinary metabolites of VOC (mVOCs) and mVOC mixtures with MetS and its components among the general adult population in the United States.

**Methods:**

A total of 5345 participants with eligible data were filtered from the 2011–2020 cycles of the National Health and Nutrition Examination Survey. Multivariate logistic regression models were applied to assess the associations of individual mVOCs with MetS and its components. The least absolute shrinkage and selection operator (LASSO) regression models were constructed to identify more relevant mVOCs. The weight quantile sum regression model was applied to further explore the links between mVOC co-exposure and MetS and its components.

**Results:**

The results indicated positive associations between multiple mVOCs and MetS, including CEMA, DHBMA, and HMPMA. CEMA was found to be positively correlated with all components of MetS. HMPMA was associated with elevated triglyceride (TG), reduced high-density lipoprotein, and fasting blood glucose (FBG) impairment; 3HPMA was associated with an elevated risk of high TG and FBG impairment; and DHBMA had positive associations with elevated TG and high blood pressure. The co-exposure of LASSO-selected mVOCs was associated with an increased risk of elevated TG, high blood pressure, and FBG impairment.

**Conclusion:**

Positive associations of certain individual urinary mVOCs and mVOC mixtures with MetS and its components were observed by utilizing multiple statistical models and large-scale national data. These findings may serve as the theoretical basis for future experimental and mechanistic studies and have important implications for public health.

**Supplementary Information:**

The online version contains supplementary material available at 10.1186/s12889-024-18198-2.

## Background

With the advancement of urbanization and industrialization, air pollution has emerged as an increasingly serious concern jeopardizing human health [1]. Diverse air pollutants exist, with volatile organic compounds (VOCs) constituting a series of low-molecular-mass carbonaceous organics characterized by easy evaporation under normal pressure and temperature and mainly originating from tobacco smoking and vehicle emissions [2, 3]. Through inhalation and dietary intake, the VOCs can directly manifest their inherent toxicological properties, leading to health problems, while their strong photochemical reactivity also allows them to be converted into other environmental pollutants, adding extra environmental pollution and indirectly causing health impairment [4, 5]. Several VOCs have already been classified as chemicals of top public health concern by the Agency for Toxic Substances and Disease Registry in its Substance Priority List [6]. Current evidence suggests that exposure to VOCs has negative impacts on multiple human systems, including the respiratory, digestive, hematology, and nervous systems [7–10]. Recent studies have further demonstrated that VOC exposure is also connected to the dysfunction of the endocrine and metabolic system. For instance, Wang et al. found that VOC exposure may cause insulin resistance and glucose homeostasis disruption, resulting in the onset of diabetes mellitus [11]. A study by Lei et al. revealed that exposure to certain VOCs was positively associated with elevated body mass index, waist circumference, and obesity risk [5]. Individual VOCs and VOC mixtures were also found to be inversely associated with bone mineral density, thereby increasing the risk of osteoporosis and fragility fractures [12].

Metabolic syndrome (MetS) represents a complex health issue characterized by a cluster of metabolic abnormalities, including abnormal glucose and lipid metabolism, hypertension, and central obesity [13]. The prevalence of MetS in the United States was estimated to be 34.7%, corresponding to approximately 1/3 of the total population, and its disease burden is continuing to increase [14]. MetS and its components are considered to be important risk factors for cardiovascular diseases, end-stage renal disease, blindness, and dementia, decreasing quality of life and overall health conditions [15, 16]. Considering the elevated disease burden and potential adverse outcomes of MetS, screening for modifiable risk factors and timely management are critical for preventing and controlling this disease [17]. MetS was once generally thought to be caused primarily by unhealthy lifestyles and behaviors, such as lack of physical activity and nutritional imbalances. However, several previous studies have uncovered that environmental pollutants, for example, organophosphate esters, cadmium, perchlorate, and thiocyanate, are also important influential factors in the occurrence and progression of MetS [18–20]. Given the evidence of the negative impacts of VOCs on the metabolic system, exposure to VOCs may also be associated with MetS and its components. However, studies on this topic remain scarce. Therefore, our study aimed to investigate the associations of individual VOC and VOC mixture exposure with MetS and its components. The findings of the current study may provide epidemiological clues for future studies focused on MetS prevention.

## Methods

### Study design and participants

**Fig. 1:**
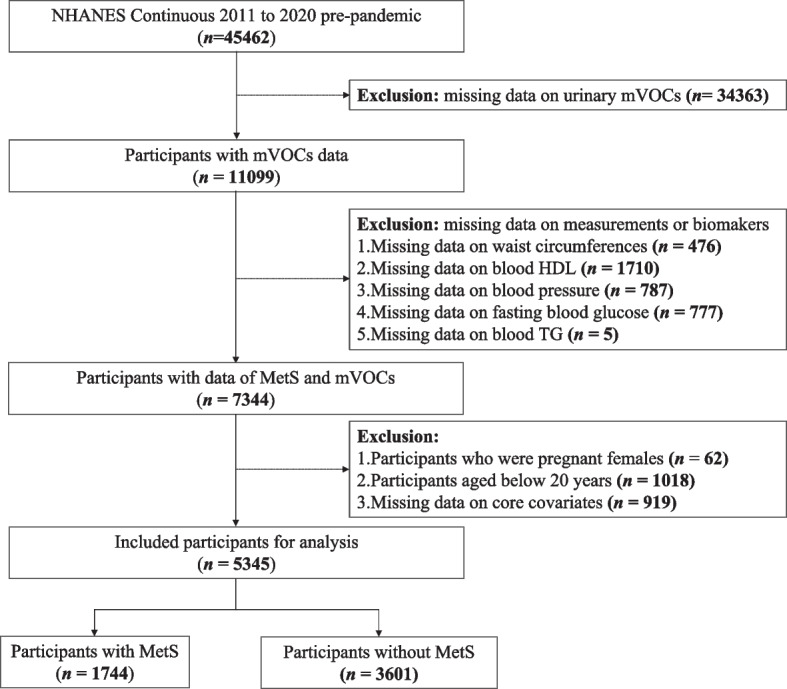
Flowchart of participants inclusion process. *Abbreviations*: NHANES, National Health and Nutrition Examination Survey; mVOCs, metabolites of volatile organic compounds; HDL, high-density lipoprotein; TG, triglyceride; MetS, metabolic syndrome

The data utilized in this cross-sectional study, including demographic features, laboratory biomarkers, physical examinations, and questionnaire data, were obtained from the National Health and Nutrition Examination Survey (NHANES). NHANES, a nationwide program of the National Center for Health Statistics (NCHS), is designed to assess the health and nutritional status of adults and children in the United States. The protocol of NHANES was approved by the NCHS Institutional Review Board, and written consent was obtained from all participants. More details on the NHANES can be found on its official website (https://www.cdc.gov/nchs/nhanes/about_nhanes.htm). For the present study, four NHANES cycles including 2011–2012, 2013–2014, 2015–2016, and 2017–2020, with a total of 45,462 participants, were collected and merged. Urinary metabolites of VOC (mVOCs) with only similar values, or missing values and values below the lower limit of detection (LLOD) exceeding 1/3 of the total population were removed in consideration of data representativeness and result robustness, leaving 15 mVOCs for analysis. The official abbreviation and LLOD for each included urinary mVOC are depicted in Table S1. To evaluate the associations of urine mVOCs with MetS and its components, participants with missing data on any of the 15 mVOCs (*n* = 34,363) or physical measurements and biomarkers related to MetS or its components (*n* = 3755) were initially excluded. Participants with missing data on core covariates (*n* = 919), those aged below 20 years (*n* = 1018), or pregnant females (*n* = 62) were further omitted. A total of 5345 participants were ultimately included in this study. The detailed participant inclusion process is depicted in Fig. 1.

### Measurement of urine metabolites of volatile organic compounds

Due to the discrepancies in VOC sources and metabolism among individuals, measuring VOCs in the air or other environmental media may not accurately illustrate the levels of individual exposure. Therefore, mVOCs were selected as biomarkers to represent the level of VOC exposure in the current study. Compared to blood mVOCs, urinary mVOCs have the advantages of long physiological half-lives and noninvasiveness [21, 22]. The participants were instructed to collect a complete void by emptying the bladder into the urine collection cup, and the volume of urine required to complete each participant’s protocol is displayed in the urine collection module. Urine samples were stored under appropriate frozen conditions (− 30 °C) until they were shipped to the National Center for Environmental Health for testing. Quantitative measurements of urinary mVOCs were performed using ultra-performance liquid chromatography coupled with electrospray tandem mass spectrometry (UPLC-ESI/MSMS) as described by Alwis et al. [21] The Acquity UPLC® HSS T3 (Part no. 186003540, 1.8 μm × 2.1 mm × 150 mm, Waters Inc.) column with 15 mM ammonium acetate and acetonitrile as the mobile phases, was used for chromatographic separation. Briefly, the eluent from the column is initially ionized using an electrospray interface to generate and transmit negative ions into the mass spectrometer. Afterwards, the comparison of relative response factors (the ratio of native analyte to stable isotope-labeled internal standard) with known standard concentrations yielded individual analyte concentrations. Detailed information on the mVOC measurements can be found on the official website (https://wwwn.cdc.gov/Nchs/Nhanes/2017-2018/P_UVOC.htm).

### Definition of metabolic syndrome and its components

The diagnostic criterion proposed by the Adult Treatment Program III of the National Cholesterol Education Program was applied as the definition of metabolic syndrome in this study [23]. Individuals with at least three of the following components were classified as MetS:

1. **Central obesity:** waist circumference ≥ 102 cm for males or ≥ 88 cm for females;
2. **Elevated triglyceride:** blood triglyceride (TG) ≥150 mg/dL or current using medication to treat elevated TG;
3. **Reduced high-density lipoprotein:** blood high-density lipoprotein (HDL) < 40 mg/dL for males, blood HDL < 50 mg/dL for females, or currently using medication for low HDL;
4. **High blood pressure:** systolic blood pressure (SBP) ≥ 130 mmHg or (and) diastolic blood pressure (DBP) ≥ 85 mmHg or currently using medication for high blood pressure.
5. **Impaired fasting blood glucose:** fasting blood glucose (FBG) ≥ 110 mg/dL or current use of medication to treat hyperglycemia.

Blood samples used for TG, HDL, and FBG testings were collected after an 8–12 fasting session. Measurements of waist circumference and blood pressure were performed by trained professionals at the mobile examination center via standardized procedures. The mean values of all three times blood pressure measurements were calculated for use in the analysis. Additional details are available on the official website of NHANES (https://wwwn.cdc.gov/nchs/nhanes/).

### Covariates

The computer-assisted personal interview (CAPI) system was utilized to reduce data entry errors and assist interviewers in defining key terms used in the questionnaire in the NHANES. Information on demographic characteristics, lifestyle, disease history, physical activity, and clinical and nutritional factors was collected from the participants using standardized questionnaires by trained interviewers with the assistance of the CAPI system. The demographic features (including age, gender, race or ethnicity, education level, marital status, and poverty-income ratio [PIR]), life behaviors (smoking status, alcohol consumption, daily total energy intake, physical activity), history of cancer, and urine creatinine were selected and set as covariates for statistical analysis based on previous studies [18, 19]. Age was divided into three groups (20–39 years, 40–59 years, and ≥ 60 years); Mexican American, other Hispanic, non-Hispanic White, non-Hispanic Black, and other races (including multi-racial) were included in the race/ethnicity category. Education condition was classified into three levels (middle school or lower, high school, and college or above). Marriage status was separated into three groups (married or living with the partner, widowed/divorced/separated, and never married). The PIR was grouped according to its quartile. The smoking status was separated into three groups (never, former smoker, and current smoker) which were determined by the questions “Have you smoked at least 100 cigarettes in your entire life?” and “Do you now smoke cigarettes?”. Self-reported alcohol consumption was obtained to divide individuals into never, 1–3 drinks per day, and ≥ 4 drinks per day. Total daily energy intake was calculated as the average value from two 24-hour dietary recall questionnaires. The physical activity status of the participants was divided into low (< 600 min/week), moderate (600–8000 min/week), and high (≥ 8000 min/week) using the metabolic equivalent of task (MET) (MET min/week) [24]. The cancer history was dichotomized as yes or no. Urine creatinine was used to correct the urinary concentrations of mVOCs, and these variables were available in the laboratory data from NHANES cycles.

### Statistical analysis

All the statistical analyses in this study were performed with the statistical packages in R software 4.3.0. Due to the complex and multi-stage probability survey design, weights were applied where appropriate in the following analyses in accordance with the NHANES analysis guidelines. Continuous variables were presented as mean (standard deviation, SD) or median (inter-quartile range, IQR), while categorical variables were described as frequencies (*n*) and weighted percentages. Values of urine mVOCs below the LLOD were replaced by an imputed fill value of (*LLOD/* $\sqrt{2}$) based on the NHANES analytic guidelines. Concentrations of all 15 urinary mVOCs were calibrated with urinary creatinine (unit: ng/mg creatinine) and naturally logarithmically transformed prior to analysis to control the concentration dilution of urine and reduce the impact of extreme values. Kruskal-Wallis test and Chi-square test were used to compare continuous and categorical variables, respectively. Multivariate logistic regression modeling was performed to explore the associations of individual urinary mVOCs with MetS and its components. Stratified analysis was used to further explore the potential subgroup-specific differences in the associations of individual urinary mVOCs with MetS. Urinary mVOCs were initially converted to categorical variables (Q1, Q2, Q3, and Q4) based on inter-quartile spacing, and the first quantile was set as the reference. Afterwards, urinary mVOCs were introduced into logistic regression models as categorical variables, with different covariates adjusted. Spearman correlation analysis was used to explore the correlations within the 15 mVOCs. Due to the expected high correlation and collinearity that usually exist among urinary mVOCs, multivariate least absolute shrinkage and selection operator (LASSO) regression was performed to avoid collinearity and determine urine mVOCs exhibiting stronger connections to MetS and its components. Finally, the selected mVOCs were included in the weighted quantile sum (WQS) regression model to explore the associations of mVOC co-exposure with MetS and its components, as well as the extent of contribution of each urinary mVOC. In the WQS analysis, 60% of the samples were randomly selected as validation datasets and 1000 bootstrap samples were used to produce stable estimates. All the statistical analyses performed in this study were two-sided, and a *P* < 0.05 was considered statistically significant.

## Results

### Characteristics of the participants

**Fig. 2:**
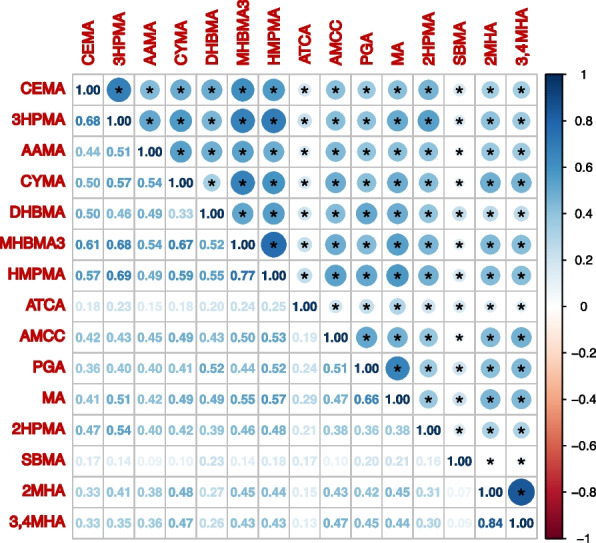
Heatmap of Spearman correlation analysis for urinary mVOCs. *Notes*: *, statistical significance (*P* < 0.05). *Abbreviations*: CEMA, N-Acetyl-S-(2-carboxyethyl)-L-cysteine; 3HPMA, N-Acetyl-S-(3-hydroxypropyl)-L-cysteine; AAMA, N-Acetyl-S-(2-carbamoylethyl)-L-cysteine; CYMA, N-Acetyl-S-(2-cyanoethyl)-L-cysteine; DHBMA, N-Acetyl-S-(3,4-dihydroxybutyl)-L-cysteine; MHBMA3, N-Acetyl-S-(4-hydroxy-2-butenyl)-L-cysteine; HMPMA, N-Acetyl-S-(3-hydroxypropyl-1-methyl)-L-cysteine; ATCA, 2-Aminothiazoline-4-carboxylic acid; AMCC, N-Acetyl-S-(N-methylcarbamoyl)-L-cysteine; PGA, Phenylglyoxylic acid; MA, Mandelic acid; 2HPMA, N-Acetyl-S-(2-hydroxypropyl)-L-cysteine; SBMA, N-Acetyl-S-(benzyl)-L-cysteine; 2MHA, 2-Methylhippuric acid; 3,4-MHA, 3- and 4-Methylhippuric acid

Among the included 5345 participants, 1744 of them were classified into the MetS group, with a weighted prevalence of 30.60%. The participants with MetS components of central obesity, elevated TG, reduced HDL, high blood pressure, and impaired FBG accounted for 57.93% (*n* = 3075), 34.39% (*n* = 1805), 27.74% (*n* = 1563), 41.26% (*n* = 2506), and 20.20% (*n* = 1210), respectively. The characteristics of the included participants are summarized in Table 1. Compared with those in the non-MetS group, individuals with MetS were more likely to be older, non-Hispanic Whites, married or lived with a partner, and had a lower education level, higher PIR, urine creatinine, waist circumference, and metabolic biomarkers (except for blood HDL, which was lower in the MetS group) (all *P* values < 0.05). Most of the participants with MetS were current smokers, alcohol drinkers, and had insufficient physical activity (all *P* values < 0.05). In addition, the proportions of hypertension, type 2 diabetes, and cancer were relatively higher in the MetS group (all *P* values < 0.05). The creatinine-corrected urinary concentrations of CEMA, 3HPMA, DHBMA, MHBMA3, and HMPMA were higher in the MetS group than in the non-MetS group (all *P* values < 0.05). The correlation coefficient matrices within 15 urinary mVOCs are illustrated in Fig. 2. Positive correlations ranging from 0.07 (2MHA and SBMA) to 0.84 (3,4MHA and 2MHA) were found between each pair of the 15 mVOCs with statistical significance (all *P* values < 0.05).

**Table 1:** Characteristics of included participants by MetS diagnosis in NHANES 2011–2020

Characteristics	Overall (*n* = 5345)	non-MetS (*n* = 3601)	MetS (*n* = 1744)	*P* value
**Weighted percentage (%)**	100	69.40	30.60	
**Gender, ** ***n*** ** (%)**				0.29^a^
Male	2733(49.59)	1854(48.94)	879(51.06)	
Female	2612(50.41)	1747(51.06)	865(48.94)	
**Age, ** ***n*** ** (%)**				< 0.01^a^
20–39 years	1791(36.29)	1481(43.90)	310(19.02)	
40–59 years	1727(35.79)	1104(33.32)	623(41.38)	
60 and above years	1827(27.92)	1016(22.77)	811(39.60)	
**Race/Ethnicity, ** ***n*** ** (%)**				< 0.05^a^
Mexican American	623(7.70)	367(7.21)	256(8.82)	
Non-Hispanic Black	1270(11.06)	853(11.09)	417(10.98)	
Non-Hispanic White	2037(66.95)	1358(66.44)	679(68.10)	
Other Hispanic	560(6.41)	372(6.69)	188(5.77)	
Other Race	855(7.89)	651(8.57)	204(6.33)	
**Education level, ** ***n*** ** (%)**				< 0.01^a^
Middle school or lower	1028(12.98)	600(11.48)	428(16.40)	
High School	2883(53.53)	1903(51.11)	980(59.01)	
College or above	1434(33.49)	1098(37.42)	336(24.59)	
**Marriage status, ** ***n*** ** (%)**				< 0.01^a^
Married or living with the partner	3190(63.34)	2127(62.62)	1063(64.97)	
Never married	1034(18.70)	799(21.69)	235(11.94)	
Widowed/Divorced/Separated	1121(17.95)	675(15.69)	446(23.09)	
**Poverty-income ratio, ** ***n*** ** (%)**				< 0.01^a^
Quartile 1	1332(17.04)	881(17.09)	451(16.95)	
Quartile 2	1328(20.26)	824(18.62)	504(23.96)	
Quartile 3	1346(27.85)	906(27.34)	440(29.02)	
Quartile 4	1339(34.85)	990(36.95)	349(30.07)	
**Smoking status, ** ***n*** ** (%)**				< 0.01^a^
Current	2082(39.04)	1313(36.53)	769(44.74)	
Former	227(4.22)	162(4.70)	65(3.14)	
Never	3036(56.73)	2126(58.77)	910(52.13)	
**Alcohol consumption, ** ***n*** ** (%)**				< 0.01^a^
Never	1722(26.89)	1082(25.48)	640(30.10)	
1–3 drink/day	1280(25.42)	869(24.34)	411(27.87)	
≥ 4 drink/day	2343(47.69)	1650(50.18)	693(42.04)	
**Physical activity, ** ***n*** ** (%)**				< 0.01^a^
Low	1984(32.89)	1182(28.33)	802(43.25)	
Moderate	2545(52.13)	1814(55.02)	731(45.57)	
High	816(14.98)	605(16.66)	211(11.18)	
**Hypertension, ** ***n*** ** (%)**				< 0.01^a^
YES	2158(34.43)	993(22.25)	1165(62.05)	
NO	3187(65.57)	2608(77.75)	579(37.95)	
**Type 2 diabetes, ** ***n*** ** (%)**				< 0.01^a^
YES	1061(16.07)	252(5.35)	809(40.40)	
NO	4284(83.93)	3349(94.65)	935(59.60)	
**History of cancer, ** *n* ** (%)**				< 0.01^a^
YES	473(9.57%)	278(7.99)	202(13.15)	
NO	4872(90.43)	3330(92.01)	1542(86.85)	
**Abdominal obesity, ** ***n*** ** (%)**				< 0.01^a^
YES	3075(57.93)	1516(43.20)	1559(91.32)	
NO	2270(42.07)	2085(56.80)	185(8.68)	
**Elevated TG, ** ***n*** ** (%)**				< 0.01^a^
YES	1805(34.39)	562(16.38)	1243(75.23)	
NO	3540(65.61)	3039(83.62)	501(24.77)	
**Reduced HDL, ** ***n*** ** (%)**				< 0.01^a^
YES	1563(27.74)	487(13.29)	1076(60.53)	
NO	3782(72.26)	3114(86.71)	668(39.47)	
**High BP, ** ***n*** ** (%)**				< 0.01^a^
YES	2506(41.26)	1146(26.38)	1360(75.02)	
NO	2839(58.74)	2455(73.62)	384(25.98)	
**Impaired FBG, ** ***n*** ** (%)**				< 0.01^a^
YES	1210(20.20)	265(6.20)	945(51.96)	
NO	4135(79.80)	3336(93.80)	799(49.04)	
WC, cm(median[IQR])	98.50[87.80,109.73]	93.00[84.40,103.50]	110.00[102.00,120.70]	< 0.01^b^
SBP, mmHg (median[IQR])	119.33[110.67,130.67]	116.67[108.67,126.00]	128.67[117.33,137.69]	< 0.01^b^
DBP, mmHg (median[IQR])	71.33[64.67,78.00]	70.33[64.00,76.67]	74.67[67.33,82.00]	< 0.01^b^
FBG, mg/dL (median[IQR])	93.00[85.00,103.00]	90.00[84.00,97.00]	104.00[92.00,124.00]	< 0.01^b^
TG (median[IQR])	115.00[79.00,176.00]	95.00[70.00,132.00]	197.00[150.00,273.33]	< 0.01^b^
HDL, mg/dL (median[IQR])	51.00[42.00,62.00]	56.00[47.00,66.00]	42.00[36.00,49.00]	< 0.01^b^
Urine creatinine, mg/dL (median[IQR])	99.00[55.00,159.00]	98.00[52.00,155.43]	102.00[61.00,165.75]	< 0.01^b^
Energy, kcal (median[IQR])	1960.15[1495.71,2545.86]	1950.00[1496.01,2562.43]	1970.80[1495.24,2515.14]	0.91^b^
CEMA, ng/mg Cr.(median[IQR])	0.96[0.61,1.61]	0.90[0.56,1.52]	1.08[0.71,1.91]	< 0.01^b^
3HPMA, ng/mg Cr.(median[IQR])	2.22[1.44,4.41]	2.12[1.40,4.38]	2.46[1.54,4.64]	< 0.01^b^
AAMA, ng/mg Cr.(median[IQR])	0.52[0.33,0.91]	0.52[0.33,0.92]	0.51[0.32,0.87]	0.28^b^
CYMA, ng/mg Cr.(median[IQR])	0.02[0.01,0.07]	0.02[0.01,0.08]	0.02[0.01,0.06]	0.45^b^
DHBMA, ng/mg Cr.(median[IQR])	3.14[2.42,4.09]	3.03[2.36,3.94]	3.39[2.57,4.39]	< 0.01^b^
MHBMA3, ng/mg Cr.(median[IQR])	0.05[0.03,0.09]	0.05[0.03,0.09]	0.05[0.03,0.10]	< 0.01^b^
HMPMA, ng/mg Cr.(median[IQR])	2.15[1.53,3.94]	2.05[1.46,3.77]	2.37[1.69,4.46]	< 0.01^b^
ATCA, ng/mg Cr.(median[IQR])	1.16[0.58,2.15]	1.15[0.59,2.11]	1.19[0.58,2.20]	0.17^b^
AMCC, ng/mg Cr.(median[IQR])	1.63[0.93,2.97]	1.64[0.93,2.93]	1.62[0.92,3.08]	0.64^b^
PGA, ng/mg Cr.(median[IQR])	2.12[1.56,2.93]	2.12[1.58,2.93]	2.13[1.54,2.89]	0.67^b^
MA, ng/mg Cr.(median[IQR])	1.35[0.97,1.99]	1.34[0.97,1.95]	1.38[0.96,2.03]	0.67^b^
2HPMA, ng/mg Cr.(median[IQR])	0.30[0.19,0.56]	0.31[0.19,0.57]	0.29[0.19,0.54]	0.48^b^
SBMA, ng/mg Cr.(median[IQR])	0.06[0.04,0.11]	0.06[0.04,0.12]	0.06[0.04,0.11]	0.96^b^
2MHA, ng/mg Cr.(median[IQR])	0.33[0.15,0.78]	0.35[0.15,0.82]	0.30[0.14,0.69]	0.10^b^
3,4MHA, ng/mg Cr.(median[IQR])	1.88[0.95,5.27]	1.94[0.94,5.51]	1.74[0.97,4.72]	0.38^b^

^a^ results of chi-square test, ^b^ results of Kruskal-Wallis test

*MetS* metabolic syndrome, *IQR* interquartile range, *TG* triglyceride, *HDL* high-density lipoprotein, *BP* blood pressure, *FBG* fasting blood glucose, *WC* waist circumference, *SBP* systolic blood pressure, *DBP* diastolic blood pressure, *Cr* creatinine, *CEMA* N-Acetyl-S-(2-carboxyethyl)-L-cysteine, *3HPMA* N-Acetyl-S-(3-hydroxypropyl)-L-cysteine, *AAMA* N-Acetyl-S-(2-carbamoylethyl)-L-cysteine, *CYMA* N-Acetyl-S-(2-cyanoethyl)-L-cysteine, *DHBMA* N-Acetyl-S-(3,4-dihydroxybutyl)-L-cysteine, *MHBMA3* N-Acetyl-S-(4-hydroxy-2-butenyl)-L-cysteine, *HMPMA* N-Acetyl-S-(3-hydroxypropyl-1-methyl)-L-cysteine, *ATCA* 2-Aminothiazoline-4-carboxylic acid, *AMCC* N-Acetyl-S-(N-methylcarbamoyl)-L-cysteine, *PGA* Phenylglyoxylic acid, *MA* Mandelic acid, *2HPMA* N-Acetyl-S-(2-hydroxypropyl)-L-cysteine, *SBMA* N-Acetyl-S-(benzyl)-L-cysteine, *2MHA* 2-Methylhippuric acid, *3,4-MHA* 3- and 4-Methylhippuric acid

### Association of individual urinary mVOC with MetS and its components

Multivariate logistic regression modeling was performed to assess the associations between single urinary mVOCs and MetS and its components. Concentrations of creatinine-corrected urinary mVOCs were initially introduced into the models as categorical variables by quantiles (Q1, Q2, Q3, and Q4). The results indicated that, after adjusting all covariates (Model 3), the risk of MetS increased by 81.00%, 48.00%, and 60.00% in response to the highest quantile (Q4) of CEMA (*OR* = 1.81, 95% *CI*: 1.44–2.28, *P*_trend_ < 0.01), DHBMA ( *OR* = 1.48, 95% *CI*: 1.09–2.02, *P*_trend_ < 0.05), and HMPMA (*OR* = 1.60, 95% *CI*: 1.20–2.13, *P*_trend_ < 0.01) when compared with the lowest quartiles (Q1). While the highest quartiles of AMCC (*OR* = 0.62, 95% *CI*: 0.45–0.85, *P*_trend_ < 0.01), PGA (*OR* = 0.65, 95% *CI*: 0.48–0.88, *P*_trend_ < 0.01), and 2MHA (*OR* = 0.61, 95% *CI*: 0.43–0.86, *P*_trend_ < 0.01) were negatively associated with the risk of MetS. The detailed results are depicted in Fig. S1. The logistic regression models were also constructed to examine the associations between individual urinary mVOCs and each component of MetS. The results illustrated that, after adjusting for all covariates (Model 3), the urinary level of CEMA was positively associated with all the components of MetS (all *P*_trend_ < 0.05); HMPMA was associated with the risk of elevated TG, reduced HDL, and FBG impairment (all *P*_trend_ < 0.05); 3HPMA was positively associated with the elevated TG and FBG impairment (all *P*_trend_ < 0.05); and DHBMA was positively connected with the elevated TG and high blood pressure (all *P*_trend_ < 0.05). However, several urinary mVOCs were also found to be inversely associated with the components of MetS. For instance, the urinary level of AAMA was negatively associated with the MetS components of central obesity, reduced HDL, and FBG impairment (all *P*_trend_ < 0.05). The detailed results of the associations between individual mVOCs and components of MetS are presented in Tables S2-S6.

### Subgroup analyses of the association between individual urinary mVOCs and MetS

Stratification analyses were performed to further explore the associations between individual urinary mVOCs and MetS in different subgroups, and the results are demonstrated in Tables S7-S8. In the highest quantile (Q4), CEMA (males: *OR* = 1.91, 95% *CI*: 1.26–2.90, *P*_trend_ < 0.05; females: *OR* = 1.72, 95% *CI*: 1.26–2.36, *P*_trend_ < 0.01) and HMPMA (males: *OR* = 1.52, 95% *CI*: 1.03–2.24, *P*_trend_ < 0.05; females: *OR* = 1.69, 95% *CI*: 1.11–2.57, *P*_trend_ < 0.01) remained to be positively associated with the increased MetS risk in both males and females when compared with the lowest quantile (Q1). However, no statistically significant association was observed between the urinary concentration of DHBMA and MetS in both gender subgroups. Furthermore, the participants were stratified by age subgroups of 20–59 years and 60 and above years. HMPMA was found to be associated with an increased risk of MetS in the highest quantile (Q4) when compared with the lowest quantile in all age subgroups (aged 20–59 years: *OR* = 1.51, 95% *CI*: 1.06–2.15, *P*_trend_ < 0.05; aged 60 and above years: *OR* = 1.82, 95% *CI*: 1.06–3.12, *P*_trend_ < 0.05). Moreover, positive associations of CEMA (Q4: *OR* = 1.98, 95% *CI*: 1.37–2.85, *P*_trend_ < 0.01), and DHBMA (Q4: *OR* = 1.68, 95% *CI*: 1.17–2.42, *P*_trend_ < 0.05) with the elevated MetS risk were observed in the subgroup of 20–59 years.

### Identification of the more relevant mVOCs to MetS and its components by LASSO regression

**Fig. 3:**
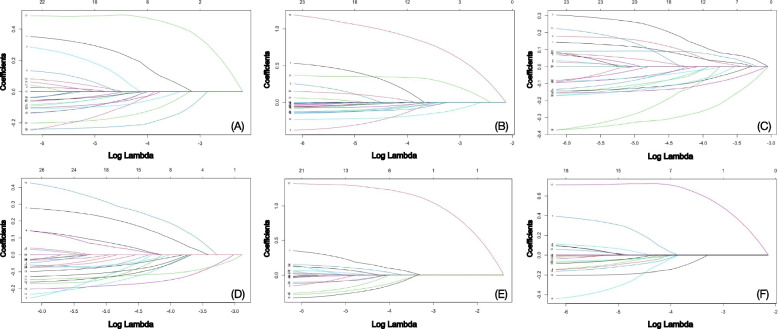
The screening pathways of the LASSO regression models. *Notes*: ln-transformed urinary concentrations of all 15 mVOCs corrected with creatinine were independent variables; MetS (**A**) and its components of central obesity (**B**), elevated TG (**C**), reduced HDL (**D**), high blood pressure (**E**), and FBG impairment (**F**) were dependent variables. The LASSO models were adjusted for all covariates including gender, age, race or ethnicity, education level, marriage status, poverty-income ratio, smoking status, alcohol drinking, daily total energy intake, physical activity, and history of cancer

**Fig. 4:**
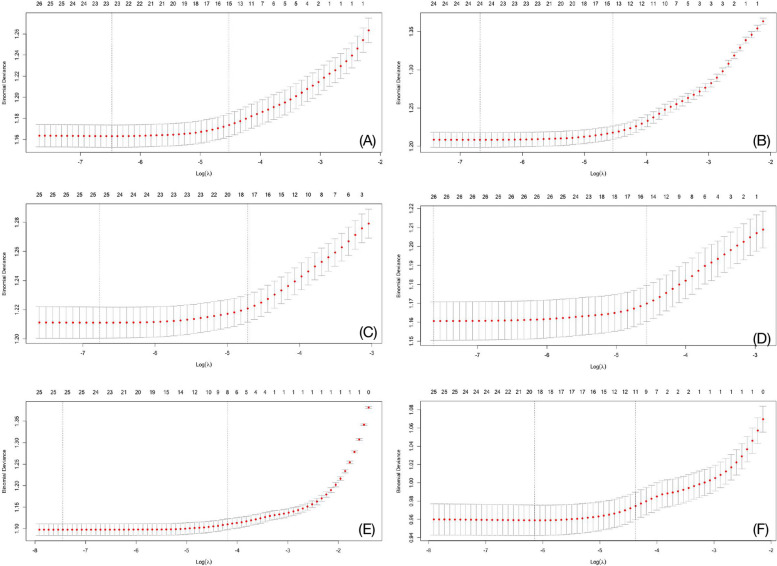
The association of the changes in binomial deviation with the penalty parameter. *Notes*: ln-transformed urinary concentrations of all 15 mVOCs corrected with creatinine were independent variables; MetS (**A**) and its components of central obesity (**B**), elevated TG (**C**), reduced HDL (**D**), high blood pressure (**E**), and FBG impairment (**F**) were dependent variables. The LASSO models were adjusted for all covariates including gender, age, race or ethnicity, education level, marriage status, poverty-income ratio, smoking status, alcohol drinking, daily total energy intake, physical activity, and history of cancer. The mean of the binomial deviation was revealed by the red colored dots, and the two dashed lines demonstrated the best λ (left line) and the simplest λ values obtained within the variance of the minimum binomial deviation (right line), respectively

Spearman correlation analysis indicated statistically significant positive correlations between every pair of the 15 urinary mVOCs, revealing the existence of high correlations and collinearity among them **(**Fig. 2**)**. Therefore, the LASSO regression modeling was performed to assess and select the mVOCs exhibiting stronger associations with MetS and its components. The associations between changes in binomial deviance and the logarithm-transformed penalty parameter (λ) in the LASSO regression models are shown in Fig. 3. By applying 10-fold cross-validation, the λ value of the simplest model obtained in a variance range of the minimum binomial deviance was estimated to be 0.0109 (log(λ) = − 4.52), 0.0107 (log(λ) = − 4.54), 0.0089(log(λ) = − 4.72), 0.0105(log(λ) = − 4.56), 0.0151(log(λ) = − 4.19), and 0.0126(log(λ) = − 4.38) obtained by using MetS and its components of central obesity, elevated TG, reduced HDL, high blood pressure, and FBG impairment as dependent variables, respectively. A total of 8, 7, 9, 7, 2, and 4 urinary mVOCs with non-zero coefficients were identified as having stronger associations with MetS and its components of central obesity, elevated TG, reduced HDL, high blood pressure, and impaired FBG, respectively (Fig. 4).

### Associations of urine mVOC co-exposure with MetS and its components

**Fig. 5:**
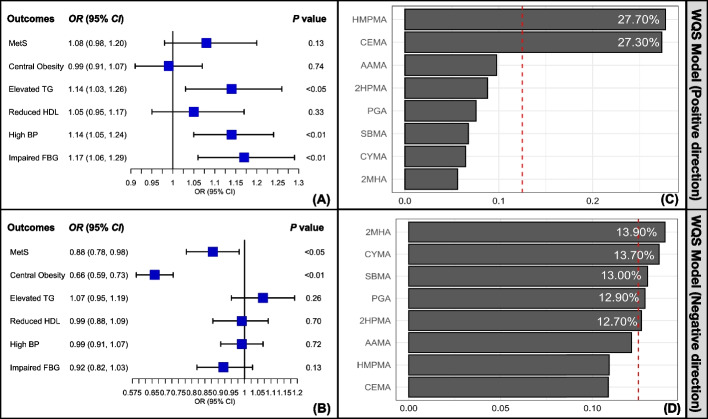
Results of the weighted quantile sum regression analyses. *Notes*: The ln-transformed urinary concentrations of the LASSO-selected mVOCs corrected with creatinine were introduced into weighted quantile sum regression models with all covariates adjusted, including gender, age, race or ethnicity, education level, marriage status, poverty-income ratio, smoking status, alcohol drinking, daily total energy intake, physical activity, and history of cancer. The weighted quantile sum regression models were constructed in both the positive direction (**A**) and negative direction (**B**). The estimate weights of each selected mVOCs in the positive association (**C**) and negative (**D**) association between mVOC mixtures and MetS were demonstrated as bar plots. *Abbreviations*: mVOCs, metabolites of volatile organic compounds; MetS, metabolic syndrome; TG, triglyceride; HDL, high-density lipoprotein; BP, blood pressure; FBG, fasting blood glucose; *OR*, odd ratio; 95% *CI*, 95% confident interval; HMPMA, N-Acetyl-S-(3-hydroxypropyl-1-methyl)-L-cysteine; CEMA, N-Acetyl-S-(2-carboxyethyl)-L-cysteine; AAMA, N-Acetyl-S-(2-carbamoylethyl)-L-cysteine; 2HPMA, N-Acetyl-S-(2-hydroxypropyl)-L-cysteine; PGA, Phenylglyoxylic acid; SBMA, N-Acetyl-S-(benzyl)-L-cysteine; CYMA, N-Acetyl-S-(2-cyanoethyl)-L-cysteine; 2MHA, 2-Methylhippuric acid

The WQS regression modeling was performed to determine the associations of co-exposure of urinary mVOCs selected by LASSO regression with MetS and its components. The WQS regression models were constructed in both the positive direction and negative direction. After adjusting for all covariates, the results of positive WQS regression analysis indicated positive associations of urinary mVOCs mixture with elevated TG (*OR* = 1.14, 95% *CI*: 1.03–1.26, *P* < 0.05), high blood pressure (*OR* = 1.14, 95% *CI*: 1.05–1.24, *P* < 0.01), and FBG impairment (*OR* = 1.17, 95% *CI*: 1.06–1.29, *P* < 0.01) (Fig. 5). 3HPMA, HMPMA, and CEMA were the three leading urinary mVOCs accounting for 12.50%, 12.00%, and 11.60% of the positive association between the mVOC mixture index and elevated TG. CEMA was the main urinary mVOC responsible for 66.40% of the positive association between mVOC mixture and high blood pressure. HMPMA was responsible for 55.90% of the positive connection between mVOC co-exposure and FBG impairment (Table S9). While in the negative model of WQS regression, negative associations were revealed between urinary mVOC mixture and MetS (*OR* = 0.88, 95% *CI*: 0.78–0.98, *P* < 0.05), as well as central obesity (*OR* = 0.66, 95% *CI*: 0.59–0.73, *P* < 0.01) (Fig. 5). 2MHA, CYMA, SBMA, PGA, and 2HPMA were responsible for a total estimated weight of 66.20% in the negative association between mVOC mixture and MetS. 2HPMA, MA, 2MHA, and SBMA accounted for a sum of 88.00% in the inverse connection between urinary mVOC co-exposure and central obesity (Table S9).

## Discussion

Exposure to several air pollutants, including particulate matter, ozone, nitrogen dioxide, and polycyclic aromatic hydrocarbons, has been found to be positively associated with the risk of MetS [25–29]. As a common type of air pollutant, the associations of VOC exposure with MetS and its components need to be further investigated. Therefore, this study utilized multiple statistical strategies to comprehensively evaluate the associations of exposure to individual VOCs and VOC mixtures with MetS and its components among the general adult population in the United States. The major findings are summarized as follows. First, 6 of the 15 urinary mVOCs were significantly linked to the risk of MetS, including both positive associations (CEMA, DHBMA, and HMPMA) and negative associations (AMCC, PGA, and 2MHA). Age- and gender-specific differences were observed in the subgroup analyses. Second, several mVOCs were found to be positively associated with the risk of MetS components. To be specific, CEMA was found to be positively correlated with the risk of all MetS components. HMPMA showed positive associations with elevated TG, reduced HDL, and FBG impairment risk; 3HPMA exhibited positive connections to the risk of elevated TG and FBG impairment; and DHBMA was positively associated with elevated TG and high blood pressure. In contrast, negative correlations were also observed between some of the mVOCs and MetS components. Third, the mVOCs that had stronger associations with MetS and its components were identified through LASSO regression analysis and subsequently introduced into WQS regression models. The results of WQS regression analyses in the positive direction indicated an increased risk of elevated TG, high blood pressure, and FBG impairment in response to elevated levels of the mVOC mixture index. CEMA was the primary contributor to the positive associations of the mVOC mixture with elevated TG and high blood pressure, while HMPMA was the largest contributor to the positive correlation between the mVOC mixture and FBG impairment. However, co-exposure to the LASSO-selected mVOCs was found to be negatively associated with the risk of MetS and central obesity. These findings are scarce evidence that can be found in previous studies, which also indicate that the associations of mVOCs with MetS and its components are sophisticated.

In the present study, CEMA, DHBMA, and HMPMA were three major urinary mVOCs identified as risk factors for MetS and its components. Notably, the urinary level of CEMA exhibited positive associations with the risk of MetS and all its components, and among the LASSO-selected mVOCs, it was identified as the major contributor to the positive correlations between the mVOC mixture exposure and elevated TG and high blood pressure. These results were consistent with several previous studies that also indicated urinary CEMA concentration was positively connected to elevated body mass index, waist circumference, and the risk of diabetes [5, 11]. Acrolein, the corresponding parent compound of CEMA [30], can cause various types of metabolic dysfunction, including MetS and its components, through multiple mechanisms. For example, the inflammatory response may be involved in the pathway of acrolein-related metabolic dysfunction. Chronic low-grade systematic inflammation is recognized as an essential common factor in the mechanism of MetS [15], while acrolein has the potential capability to activate the NF-κB pathway and serve as a pro-inflammatory mediator in umbilical vein endothelial cells [31]. Conklin et al. reported that acrolein could alter the hepatic gene expressions involving lipid synthesis, trafficking, and acute phase response stimulation in experimental mice, causing lipid metabolic abnormalities [32]. Moreover, studies have revealed that acrolein causes structural and functional alternations in the cardiovascular system, resulting in an increase in heart rate and high blood pressure [33]. In stratified analyses, findings of the positive associations between the urinary concentration of CEMA and increased risk of MetS were consistent in both the male and female subgroups. While in age subgroups, a more significant positive association was found in the subgroup of 20–59 years than in the subgroup of 60 years and above. The potential reasons for this difference may be the effects of altered CEMA exposure levels and higher MetS prevalence due to poor health status among older adults [3]. In addition, 3HPMA is considered to be another metabolite generated from acrolein [34], it was also found to be associated with an increased risk of elevated TG, and FBG impairment in the current study. The full mechanisms through which acrolein causes metabolic impairment remain unclear, and additional experimental studies are needed in the future.

The present study showed that HMPMA was positively associated with the risk of MetS and its components of elevated TG, reduced HDL, and FBG impairment, and it contributed the most to the positive relationship between the mVOC mixture and FBG impairment. The corresponding parent compound of HMPMA is crotonaldehyde, which is highly toxic and environmentally ubiquitous (e.g., found in various food products including fish, meat and fruits) [35]. It can also be exposed from tobacco smoke and the chemical processing industry [36]. Zhang et al. found that crotonaldehyde exposure can disrupt liver mitochondrial energy metabolism in mice, trigger oxidative stress, activate mitochondrial-mediated caspase, promote apoptosis, and cause hepatic dysfunction [37]. This can provide a possible explanation for the positive associations between HMPMA and elevated TG and reduced HDL, by the fact that liver is the central organ responsible for lipid homeostasis [38]. Extensive studies have also shown that crotonaldehyde can activate oxidative stress and inflammation, causing renal diseases and cardiovascular diseases [39]. Furthermore, stratified analyses revealed that the positive association between urinary concentration of HMPMA and elevated MetS risk was consistent in both gender and age subgroups, further demonstrating that the results were robust. However, studies focusing on the effects of crotonaldehyde exposure on human metabolic health remain rare. Thus, the specific mechanisms need to be further explored in future experiment studies.

DHBMA was another mVOC found to be positively associated with the risk of MetS and its components of elevated TG and high blood pressure. The corresponding parent compound of DHBMA is 1,3-butadiene (BD), which can be exposed from synthetic rubber production, petroleum processing, and combustion sources (e.g., vehicle exhaustion and cigarette smoking) [40]. BD was found to induce a premature attenuation of steroidogenesis, affecting normal lipid metabolism, and thereby causing abnormal lipid profiles [40]. McGraw et al. found exposure to BD metabolites was significantly linked to elevated SBP and endothelial dysfunction, and DHBMA was one of the most important metabolites of the group in relation to SBP and DBP, as indicated by a higher posterior inclusion probability in the Bayesian kernel machine regression model [41]. Previous experimental studies also revealed that BD exposure may trigger oxidative stress, induce the elevation of reactive oxygen species, and activate the mitochondrial apoptotic pathway [42, 43]. Similarly, several epidemiological studies further found positive associations of BD exposure with increased oxidative stress markers, vascular endothelial dysfunction, and several cardiovascular risk factors among the young population in China [40, 44, 45]. These prior studies provided possible explanations for the observed results in the current study, as the above-mentioned alternations were involved in the pathogenic mechanisms of MetS [46].

Some of the mVOCs were found to be negatively associated with MetS and its components. This negative association may be the consequence of interference from obesity. Unexpectedly, a total of 11 mVOCs were found to be inversely related to central obesity. This finding was similar to the results of a recent study by Lei et al. [5] Prior studies have indicated that most of the VOCs would increase the risk of cancer, gastrointestinal diseases, and other wasting diseases [47]. In the current study, a higher weighted percentage of cancer history was found in the MetS group, and weight loss is a major symptom of different types of cancer. In addition, exposure to VOCs is also associated with inflammatory bowel disease that usually causes nutritional imbalance, then leading to weight loss [48]. Therefore, this particular protective effect may actually be a negative impact on human health. However, this phenomenon and its underlying mechanisms deserve further investigation and demonstration in future studies.

The effects of exposure to VOC mixtures on human health is a more realistic issue, as we are often exposed to multiple types of VOCs [49]. Interestingly, co-exposure to mVOCs exhibited a negative association with the MetS risk in this study. MetS is a comprehensive metabolic disease that consists of various metabolic disorders, and the effects of VOC mixtures on different kinds of metabolic dysfunction are not always in the same direction. Our results indicated that mVOC co-exposure was negatively associated with central obesity while positively connected to elevated TG, high blood pressure, and FBG impairment. Moreover, the effects of different kinds of mVOCs are also not always preserved [11]. It was possible that the negative effects of the potential effects of several of the mVOCs offset the positive effects of the other mVOCs, ultimately resulting in a negative association.

This cross-sectional study has several strengths. First, the associations of mVOCs with MetS and its components were explored based on a large and nationwide representative sample size from the NHANES. Second, both individual urinary mVOCs and their mixture were evaluated in the current study, providing robust clues for mechanism research in the future. However, the current results should be treated with caution due to several inevitable limitations. First, this study was cross-sectional designed, which prevented corroboration and causality. Second, urinary mVOC concentrations were used to indicate the levels of VOC exposure. Although it had its own advantages of long physiological half-lives and non-invasion, the potential time-lag between biological monitoring and MetS should not be ignored. Last, various covariates were introduced into the statistical models to reduce their impact on the observed results, and subgroup analyses were performed to ensure that these findings were reliable across different scenarios. However, the impact of other unknown covariates should not be neglected.

## Conclusions

In conclusion, the current study found that exposure to certain urinary mVOCs and mVOC mixtures were positively associated with MetS and its components. Of note, CEMA was identified as a significant risk factor for MetS and all of its components. These findings suggested that exposure to specific mVOCs was significantly associated with an increased risk of MetS and its components. Further prospective studies and mechanistic studies are needed to validate the results of this study.

### Supplementary Information

**Supplementary Material 1.**

## Data Availability

The data utilized in this manuscript could be obtained from the official website of NHANES (https://www.cdc.gov/nchs/nhanes/).

## Abbreviations

VOCs: Volatile organic compounds
MetS: Metabolic syndrome
NHANES: National health and nutrition examination survey
NCHS: National center for health statistics
mVOCs: Metabolites of VOC
LLOD: Lower limit of detection
TG: Triglyceride
HDL: High-density lipoprotein
SBP: Systolic blood pressure
DBP: Diastolic blood pressure
FBG: Fasting blood glucose
CAPI: Computer-assisted personal interview
PIR: Poverty-income ratio
MET: Metabolic equivalent of task
LASSO: Least absolute shrinkage and selection operator
WQS: Weighted quantile sum
BD: 1,3-Butadiene

## References

[1] Orru H, Ebi KL, Forsberg B (2017). The interplay of climate change and air pollution on health. Curr Environ Health Rep.

[2] Inamdar AA, Morath S, Bennett JW (2020). Fungal volatile organic compounds: more than just a funky smell?. Ann Rev Microbiol.

[3] Konkle SL, Zierold KM, Taylor KC, Riggs DW, Bhatnagar A (2020). National secular trends in ambient air volatile organic compound levels and biomarkers of exposure in the United States. Environ Res.

[4] Hanna GB, Boshier PR, Markar SR, Romano A (2019). Accuracy and Methodologic challenges of volatile organic compound-based exhaled breath tests for Cancer diagnosis: a systematic review and Meta-analysis. JAMA Oncol.

[5] Lei T, Qian H, Yang J, Hu Y (2023). The association analysis between exposure to volatile organic chemicals and obesity in the general USA population: a cross-sectional study from NHANES program. Chemosphere..

[6] Agency for Toxic Substances and Disease Registry: ATSDR’s substance priority list. https://www.atsdr.cdc.gov/spl/ (2022). Accessed 30 Jan 2024.

[7] Wang Y, Han X, Li J, Zhang L, Liu Y, Jin R (2023). Associations between the compositional patterns of blood volatile organic compounds and chronic respiratory diseases and ages at onset in NHANES 2003-2012. Chemosphere..

[8] Wahlang B, Gao H, Rai SN, Keith RJ, McClain CJ, Srivastava S (2023). Associations between residential volatile organic compound exposures and liver injury markers: the role of biological sex and race. Environ Res.

[9] Vaughan Watson C, Naik S, Lewin M, Ragin-Wilson A, Irvin-Barnwell E (2021). Associations between select blood VOCs and hematological measures in NHANES 2005-2010. J Expo Sci Environ Epidemiol.

[10] Annavarapu RN, Kathi S (2016). Cognitive disorders in children associated with urban vehicular emissions. Environ Pollut.

[11] Wang X, He W, Wu X, Song X, Yang X, Zhang G, et al. Exposure to volatile organic compounds is a risk factor for diabetes: a cross-sectional study. Chemosphere. 2023;139424. 10.1016/j.chemosphere.2023.139424 37419158

[12] Zhou HL, Su GH, Zhang RY, Di DS, Wang Q (2022). Association of volatile organic compounds co-exposure with bone health indicators and potential mediators. Chemosphere..

[13] Åberg F, Byrne CD, Pirola CJ, Männistö V, Sookoian S (2023). Alcohol consumption and metabolic syndrome: clinical and epidemiological impact on liver disease. J Hepatol.

[14] Hirode G, Wong RJ (2020). Trends in the prevalence of metabolic syndrome in the United States, 2011-2016. JAMA..

[15] Ambroselli D, Masciulli F, Romano E, Catanzaro G, Besharat ZM, Massari MC (2023). New advances in metabolic syndrome, from prevention to treatment: the role of diet and food. Nutrients..

[16] Silveira Rossi JL, Barbalho SM, Reverete de Araujo R, Bechara MD, Sloan KP, Sloan LA (2022). Metabolic syndrome and cardiovascular diseases: going beyond traditional risk factors. Diabetes Metab Res Rev.

[17] Ferriani LO, Silva DA, Molina MDCB, Mill JG, Brunoni AR, da Fonseca MJM (2023). Depression is a risk factor for metabolic syndrome: results from the ELSA-Brasil cohort study. J Psychiatr Res.

[18] Luo K, Zhang R, Aimuzi R, Wang Y, Nian M, Zhang J (2020). Exposure to organophosphate esters and metabolic syndrome in adults. Environ Int.

[19] Guo X, Wu B, Hu W, Wang X, Su W, Meng J (2023). Associations of perchlorate, nitrate, and thiocyanate with metabolic syndrome and its components among US adults: a cross-sectional study from NHANES. Sci Total Environ.

[20] Xing W, Wang L, Gu W, Liang M, Wang Z, Fan D (2023). Association of blood cadmium and metabolic syndrome: a cross-sectional analysis of National Health and nutrition examination survey 2017-2020. Environ Sci Pollut Res Int.

[21] Alwis KU, Blount BC, Britt AS, Patel D, Ashley DL (2012). Simultaneous analysis of 28 urinary VOC metabolites using ultra high performance liquid chromatography coupled with electrospray ionization tandem mass spectrometry (UPLC-ESI/MSMS). Anal Chim Acta.

[22] Ding X, Zhang Y, Zhang Y, Ding X, Zhang H, Cao T (2022). Modular assembly of MXene frameworks for noninvasive disease diagnosis via urinary volatiles. ACS Nano.

[23] Grundy SM, Brewer HB, Cleeman JI, Smith SC, Lenfant C (2004). American Heart Association, et al. definition of metabolic syndrome: report of the National Heart, Lung, and Blood Institute/American Heart Association conference on scientific issues related to definition. Circulation..

[24] Kyu HH, Bachman VF, Alexander LT, Mumford JE, Afshin A, Estep K (2016). Physical activity and risk of breast cancer, colon cancer, diabetes, ischemic heart disease, and ischemic stroke events: systematic review and dose-response meta-analysis for the global burden of disease study 2013. BMJ..

[25] Wei Y, Zhang JJ, Li Z, Gow A, Chung KF, Hu M (2016). Chronic exposure to air pollution particles increases the risk of obesity and metabolic syndrome: findings from a natural experiment in Beijing. FASEB J.

[26] Li W, Chen D, Peng Y (2023). Association of polycyclic aromatic hydrocarbons with systemic inflammation and metabolic syndrome and its components. Obesity (Silver Spring).

[27] Poursafa P, Kamali Z, Fraszczyk E, Boezen HM, Vaez A, Snieder H (2022). DNA methylation: a potential mediator between air pollution and metabolic syndrome. Clin Epigenetics.

[28] Han S, Zhang F, Yu H (2022). Systemic inflammation accelerates the adverse effects of air pollution on metabolic syndrome: findings from the China health and retirement longitudinal study (CHARLS). Environ Res.

[29] Zhang JS, Gui ZH, Zou ZY (2021). Long-term exposure to ambient air pollution and metabolic syndrome in children and adolescents: a national cross-sectional study in China. Environ Int.

[30] Alfarhan M, Jafari E, Narayanan SP (2020). Acrolein: a potential mediator of oxidative damage in diabetic retinopathy. Biomolecules..

[31] Moghe A, Ghare S, Lamoreau B, Mohammad M, Barve S, McClain C (2015). Molecular mechanisms of acrolein toxicity: relevance to human disease. Toxicol Sci.

[32] Conklin DJ, Prough RA, Juvan P, Rezen T, Rozman D, Haberzettl P (2011). Acrolein-induced dyslipidemia and acute-phase response are independent of HMG-CoA reductase. Mol Nutr Food Res.

[33] Conklin DJ, Bhatnagar A, Cowley HR, Johnson GH, Wiechmann RJ, Sayre LM (2006). Acrolein generation stimulates hypercontraction in isolated human blood vessels. Toxicol Appl Pharmacol.

[34] Alwis KU, deCastro BR, Morrow JC, Blount BC (2015). Acrolein Exposure in U.S. Tobacco Smokers and Non-Tobacco Users: NHANES 2005-2006. Environ Health Perspect.

[35] Carmella SG, Chen M, Zarth A, Hecht SS (2013). High throughput liquid chromatography-tandem mass spectrometry assay for mercapturic acids of acrolein and crotonaldehyde in cigarette smokers’ urine. J Chromatogr B Analyt Technol Biomed Life Sci.

[36] Jin L, Jagatheesan G, Lynch J, Guo L, Conklin DJ (2020). Crotonaldehyde-induced vascular relaxation and toxicity: role of endothelium and transient receptor potential ankyrin-1 (TRPA1). Toxicol Appl Pharmacol.

[37] Zhang S, Zhang B, Zhang Q, Zhang Z (2021). Crotonaldehyde exposure induces liver dysfunction and mitochondrial energy metabolism disorder in rats. Toxicol Mech Methods.

[38] Alves-Bezerra M, Cohen DE (2017). Triglyceride metabolism in the liver. Compr Physiol.

[39] Zhang B, Li S, Men J, Peng C, Shao H, Zhang Z (2019). Long-term exposure to crotonaldehyde causes heart and kidney dysfunction through induction of inflammatory and oxidative damage in male Wistar rats. Toxicol Mech Methods.

[40] Lin CY, Lee HL, Jung WT, Sung FC, Su TC (2020). The association between urinary levels of 1,3-butadiene metabolites, cardiovascular risk factors, microparticles, and oxidative stress products in adolescents and young adults. J Hazard Mater.

[41] McGraw KE, Riggs DW, Rai S (2021). Exposure to volatile organic compounds - acrolein, 1,3-butadiene, and crotonaldehyde - is associated with vascular dysfunction. Environ Res.

[42] Yadavilli S, Martinez-Ceballos E, Snowden-Aikens J (2007). Diepoxybutane activates the mitochondrial apoptotic pathway and mediates apoptosis in human lymphoblasts through oxidative stress. Toxicol in Vitro.

[43] Kennedy CH, Catallo WJ, Wilson VL, Mitchell JB (2009). Combustion products of 1,3-butadiene inhibit catalase activity and induce expression of oxidative DNA damage repair enzymes in human bronchial epithelial cells. Cell Biol Toxicol.

[44] Song W, Han Q, Wan Y (2022). Repeated measurements of 21 urinary metabolites of volatile organic compounds and their associations with three selected oxidative stress biomarkers in 0-7-year-old healthy children from south and Central China. Chemosphere..

[45] Yan M, Zhu H, Luo H, Zhang T, Sun H, Kannan K (2023). Daily exposure to environmental volatile organic compounds triggers oxidative damage: evidence from a large-scale survey in China. Environ Sci Technol.

[46] Cai M, Li S, Cai K, Du X, Han J, Hu J. Empowering mitochondrial metabolism: exploring L-lactate supplementation as a promising therapeutic approach for metabolic syndrome. Metabolism. 2024; 10.1016/j.metabol.2024.155787. 10.1016/j.metabol.2024.155787 38215964

[47] Rondanelli M, Perdoni F, Infantino V, Faliva MA, Peroni G, Iannello G (2019). Volatile organic compounds as biomarkers of gastrointestinal diseases and nutritional status. J Anal Methods Chem.

[48] Smolinska A, Bodelier AG, Dallinga JW, Masclee AA, Jonkers DM, van Schooten FJ (2017). The potential of volatile organic compounds for the detection of active disease in patients with ulcerative colitis. Aliment Pharmacol Ther.

[49] Zhu L, Shen D, Luo KH (2020). A critical review on VOCs adsorption by different porous materials: species, mechanisms and modification methods. J Hazard Mater.

