# Supplementary material for: Association of volatile organic compound exposure with metabolic syndrome and its components: a nationwide cross-sectional study
Source: BMC Public Health. 2024 Mar 2;24:671. doi: 10.1186/s12889-024-18198-2 (PMC10909266; doi:10.1186/s12889-024-18198-2)
Supplement: Supplementary file 1 — Supplementary Material 1. [file 12889_2024_18198_MOESM1_ESM.docx]

**Supplementary Materials**

**Table.S1** The official abbreviation and lower limit of detection of urinary mVOCs 2

**Table.S2** Association between urine mVOCs and central obesity 3

**Table.S3** Association between urine mVOCs and elevated TG 5

**Table.S4** Association between urine mVOCs and reduced HDL 7

**Table.S5** Association between urine mVOCs and high BP 9

**Table.S6** Association between urine mVOCs and impaired FBG 11

**Table.S7** Association between urine mVOCs and MetS stratified by gender subgroups 13

**Table.S8** Association between urine mVOCs and MetS stratified by age subgroups 15

**Table.S9** Estimated weight of each LASSO-selected mVOC in WQS regression models 17

**Figure.S1** Forest plot of association between urinary mVOCs and MetS in multivariate logistic regression analysis 18

**Table.S1** The official abbreviation and lower limit of detection of urinary mVOCs

| **Parent compound** | **mVOCs detected in urine** | **Abbreviation** | **LLOD** |
| --- | --- | --- | --- |
| Acrolein | N-Acetyl-S-(2-carboxyethyl)-L-cysteine | CEMA | 6.96 |
|  | N-Acetyl-S-(3-hydroxypropyl)-L-cysteine | 3HPMA | 13.00 |
| Acrylamide | N-Acetyl-S-(2-carbamoylethyl)-L-cysteine | AAMA | 2.20 |
| Acrylonitrile | N-Acetyl-S-(2-cyanoethyl)-L-cysteine | CYMA | 0.50 |
| 1,3-Butadiene | N-Acetyl-S-(3,4-dihydroxybutyl)-L-cysteine | DHBMA | 5.25 |
|  | N-Acetyl-S-(4-hydroxy-2-butenyl)-L-cysteine | MHBMA3 | 0.60 |
| Crotonaldehyde | N-Acetyl-S-(3-hydroxypropyl-1-methyl)-L-cysteine | HMPMA | 1.70 |
| Cyanide | 2-Aminothiazoline-4-carboxylic acid | ATCA | 15.00 |
| N, N- Dimethylformamide, methyl isocyanate | N-Acetyl-S-(N-methylcarbamoyl)-L-cysteine | AMCC | 6.26 |
| Ethylbenzene, styrene | Phenylglyoxylic acid | PGA | 12.00 |
| Styrene, ethylbenzene | Mandelic acid | MA | 12.00 |
| Propylene oxide | N-Acetyl-S-(2-hydroxypropyl)-L-cysteine | 2HPMA | 5.30 |
| Toluene, benzyl alcohol | N-Acetyl-S-(benzyl)-L-cysteine | SBMA | 0.50 |
| Xylene | 2-Methylhippuric acid | 2MHA | 5.00 |
|  | 3- and 4-Methylhippuric acid | 3,4-MHA | 8.00 |

***Abbreviations:*** mVOCs, metabolites of volatile organic compounds; LLOD, lower limit of detection.

**Table.S2** Association between urine mVOCs and **central obesity**

| **Urinary mVOCs** | **Model 1** | | | **Model 2** | | | **Model 3** | | |
| --- | --- | --- | --- | --- | --- | --- | --- | --- | --- |
|  | ***OR*** | **95% CI** | ***P* value** | ***OR*** | **95% CI** | ***P* value** | ***OR*** | **95% CI** | ***P* value** |
| **CEMA** | —— | —— | —— | —— | —— | —— | —— | —— | —— |
| Q2 | 1.48 | 1.19, 1.85 | **<0.01** | 1.54 | 1.20, 1.98 | **<0.01** | 1.53 | 1.20, 1.96 | **<0.01** |
| Q3 | 1.53 | 1.24, 1.89 | **<0.01** | 1.41 | 1.12, 1.77 | **<0.01** | 1.38 | 1.10, 1.74 | **<0.01** |
| Q4 | 1.63 | 1.36, 1.97 | **<0.01** | 1.37 | 1.11, 1.69 | **<0.01** | 1.33 | 1.07, 1.66 | **<0.05** |
| *P*_trend_ | **<0.01** | | | **<0.05** | | | **<0.05** | | |
| **3HPMA** | —— | —— | —— | —— | —— | —— | —— | —— | —— |
| Q2 | 0.99 | 0.77, 1.27 | >0.90 | 1.11 | 0.85, 1.44 | 0.40 | 1.13 | 0.88, 1.46 | 0.30 |
| Q3 | 0.97 | 0.74, 1.25 | 0.80 | 1.07 | 0.80, 1.42 | 0.70 | 1.05 | 0.80, 1.39 | 0.70 |
| Q4 | 0.79 | 0.63, 1.00 | **<0.05** | 0.82 | 0.65, 1.03 | 0.08 | 0.75 | 0.60, 0.94 | **<0.05** |
| *P*_trend_ | 0.06 | | | 0.10 | | | **<0.05** | | |
| **AAMA** | —— | —— | —— | —— | —— | —— | —— | —— | —— |
| Q2 | 1.14 | 0.93, 1.40 | 0.20 | 1.03 | 0.82, 1.30 | 0.80 | 1.03 | 0.82, 1.29 | 0.80 |
| Q3 | 1.17 | 0.95, 1.44 | 0.14 | 1.04 | 0.84, 1.29 | 0.70 | 1.02 | 0.82, 1.27 | 0.90 |
| Q4 | 0.87 | 0.68, 1.12 | 0.30 | 0.75 | 0.60, 0.94 | **<0.05** | 0.69 | 0.54, 0.89 | **<0.01** |
| *P*_trend_ | 0.30 | | | **<0.05** | | | **<0.01** | | |
| **CYMA** | —— | —— | —— | —— | —— | —— | —— | —— | —— |
| Q2 | 0.82 | 0.63, 1.08 | 0.20 | 0.78 | 0.59, 1.04 | 0.08 | 0.78 | 0.59, 1.05 | 0.10 |
| Q3 | 0.9 | 0.68, 1.18 | 0.40 | 0.81 | 0.61, 1.09 | 0.2 | 0.79 | 0.59, 1.07 | 0.12 |
| Q4 | 0.69 | 0.52, 0.92 | **<0.05** | 0.64 | 0.49, 0.83 | **<0.01** | 0.53 | 0.39, 0.72 | **<0.01** |
| *P*_trend_ | **<0.05** | | | **<0.01** | | | **<0.01** | | |
| **DHBMA** | —— | —— | —— | —— | —— | —— | —— | —— | —— |
| Q2 | 1.49 | 1.22, 1.83 | **<0.01** | 1.25 | 1.02, 1.54 | **<0.05** | 1.26 | 1.02, 1.55 | **<0.05** |
| Q3 | 1.47 | 1.18, 1.82 | **<0.01** | 1.07 | 0.85, 1.36 | 0.60 | 1.09 | 0.86, 1.39 | 0.50 |
| Q4 | 1.63 | 1.32, 2.02 | **<0.01** | 1.01 | 0.80, 1.27 | >0.90 | 1.01 | 0.78, 1.31 | >0.90 |
| *P*_trend_ | **<0.01** | | | 0.70 | | | 0.80 | | |
| **MHBMA3** | —— | —— | —— | —— | —— | —— | —— | —— | —— |
| Q2 | 1.09 | 0.83, 1.44 | 0.50 | 0.96 | 0.73, 1.27 | 0.80 | 0.97 | 0.73, 1.29 | 0.80 |
| Q3 | 1.00 | 0.81, 1.23 | >0.90 | 0.82 | 0.65, 1.02 | 0.07 | 0.81 | 0.64, 1.02 | 0.07 |
| Q4 | 0.90 | 0.70, 1.16 | 0.40 | 0.75 | 0.60, 0.94 | **<0.05** | 0.68 | 0.54, 0.85 | **<0.01** |
| *P*_trend_ | 0.30 | | | **<0.01** | | | **<0.01** | | |
| **HMPMA** | —— | —— | —— | —— | —— | —— | —— | —— | —— |
| Q2 | 1.28 | 0.99, 1.66 | 0.06 | 1.03 | 0.80, 1.34 | 0.80 | 1.02 | 0.79, 1.32 | 0.9 |
| Q3 | 1.47 | 1.08, 2.00 | **<0.05** | 1.14 | 0.83, 1.58 | 0.40 | 1.11 | 0.81, 1.52 | 0.5 |
| Q4 | 0.98 | 0.76, 1.27 | 0.90 | 0.73 | 0.57, 0.94 | **<0.05** | 0.65 | 0.49, 0.86 | **<0.01** |
| *P*_trend_ | 0.90 | | | 0.06 | | | **<0.05** | | |
| **ATCA** | —— | —— | —— | —— | —— | —— | —— | —— | —— |
| Q2 | 0.94 | 0.75, 1.18 | 0.60 | 0.73 | 0.57, 0.94 | **<0.05** | 0.74 | 0.57, 0.95 | **<0.05** |
| Q3 | 1.22 | 1.00, 1.48 | **<0.05** | 0.78 | 0.63, 0.98 | **<0.05** | 0.77 | 0.62, 0.96 | **<0.05** |
| Q4 | 1.57 | 1.26, 1.95 | **<0.01** | 0.83 | 0.64, 1.08 | 0.20 | 0.84 | 0.65, 1.09 | 0.20 |
| *P*_trend_ | **<0.01** | | | 0.20 | | | 0.20 | | |
| **AMCC** | —— | —— | —— | —— | —— | —— | —— | —— | —— |
| Q2 | 1.19 | 0.91, 1.56 | 0.20 | 0.94 | 0.70, 1.26 | 0.70 | 0.91 | 0.69, 1.20 | 0.50 |
| Q3 | 1.07 | 0.83, 1.39 | 0.60 | 0.74 | 0.57, 0.97 | **<0.05** | 0.68 | 0.52, 0.91 | **<0.05** |
| Q4 | 1.16 | 0.93, 1.44 | 0.20 | 0.67 | 0.53, 0.86 | **<0.01** | 0.58 | 0.44, 0.77 | **<0.01** |
| *P*_trend_ | 0.30 | | | **<0.01** | | | **<0.01** | | |
| **PGA** | —— | —— | —— | —— | —— | —— | —— | —— | —— |
| Q2 | 0.98 | 0.75, 1.27 | 0.90 | 0.80 | 0.61, 1.06 | 0.11 | 0.81 | 0.62, 1.07 | 0.13 |
| Q3 | 1.07 | 0.84, 1.35 | 0.60 | 0.76 | 0.60, 0.97 | **<0.05** | 0.77 | 0.60, 0.98 | **<0.05** |
| Q4 | 1.01 | 0.77, 1.31 | >0.90 | 0.63 | 0.48, 0.82 | **<0.01** | 0.62 | 0.46, 0.83 | **<0.01** |
| *P*_trend_ | 0.80 | | | **<0.01** | | | **<0.01** | | |
| **MA** | —— | —— | —— | —— | —— | —— | —— | —— | —— |
| Q2 | 1.01 | 0.80, 1.26 | >0.90 | 0.98 | 0.75, 1.28 | 0.90 | 0.98 | 0.75, 1.29 | 0.90 |
| Q3 | 1.05 | 0.85, 1.31 | 0.60 | 0.93 | 0.72, 1.19 | 0.60 | 0.91 | 0.70, 1.17 | 0.40 |
| Q4 | 0.90 | 0.72, 1.14 | 0.40 | 0.71 | 0.55, 0.91 | **<0.01** | 0.67 | 0.51, 0.89 | **<0.01** |
| *P*_trend_ | 0.50 | | | **<0.01** | | | **<0.01** | | |
| **2HPMA** | —— | —— | —— | —— | —— | —— | —— | —— | —— |
| Q2 | 0.93 | 0.75, 1.16 | 0.5 | 0.84 | 0.66, 1.09 | 0.2 | 0.83 | 0.64, 1.08 | 0.20 |
| Q3 | 0.79 | 0.62, 1.00 | 0.051 | 0.74 | 0.57, 0.97 | **<0.05** | 0.71 | 0.55, 0.93 | **<0.05** |
| Q4 | 0.74 | 0.59, 0.93 | **<0.05** | 0.61 | 0.49, 0.76 | **<0.01** | 0.57 | 0.46, 0.70 | **<0.01** |
| *P*_trend_ | **<0.01** | | | **<0.01** | | | **<0.01** | | |
| **SBMA** | —— | —— | —— | —— | —— | —— | —— | —— | —— |
| Q2 | 1.08 | 0.86, 1.35 | 0.50 | 0.97 | 0.77, 1.22 | 0.80 | 0.96 | 0.76, 1.21 | 0.70 |
| Q3 | 1.16 | 0.93, 1.45 | 0.20 | 0.89 | 0.69, 1.15 | 0.40 | 0.87 | 0.67, 1.14 | 0.30 |
| Q4 | 1.17 | 0.93, 1.48 | 0.20 | 0.81 | 0.64, 1.04 | 0.10 | 0.82 | 0.64, 1.05 | 0.12 |
| *P*_trend_ | 0.14 | | | 0.08 | | | 0.09 | | |
| **2MHA** | —— | —— | —— | —— | —— | —— | —— | —— | —— |
| Q2 | 0.78 | 0.61, 1.00 | **<0.05** | 0.74 | 0.57, 0.95 | **<0.05** | 0.75 | 0.58, 0.96 | **<0.05** |
| Q3 | 0.85 | 0.65, 1.11 | 0.2 | 0.81 | 0.62, 1.06 | 0.13 | 0.8 | 0.60, 1.05 | 0.1 |
| Q4 | 0.71 | 0.57, 0.88 | **<0.01** | 0.6 | 0.48, 0.75 | **<0.01** | 0.57 | 0.45, 0.73 | **<0.01** |
| *P*_trend_ | **<0.05** | | | **<0.01** | | | **<0.01** | | |
| **3,4MHA** | —— | —— | —— | —— | —— | —— | —— | —— | —— |
| Q2 | 0.98 | 0.73, 1.31 | 0.9 | 0.83 | 0.62, 1.11 | 0.2 | 0.86 | 0.65, 1.15 | 0.30 |
| Q3 | 0.9 | 0.69, 1.16 | 0.4 | 0.76 | 0.58, 1.00 | **<0.05** | 0.77 | 0.59, 1.01 | 0.06 |
| Q4 | 0.88 | 0.70, 1.11 | 0.3 | 0.67 | 0.53, 0.86 | **<0.01** | 0.65 | 0.49, 0.85 | **<0.01** |
| *P*_trend_ | 0.2 | | | **<0.01** | | | **<0.01** | | |

***Notes:*** Urinary mVOCs were introduced into logistic regression models as categorical variables. All results were presented as OR, 95% *CI*, *P* value, and *P*_trend_. **Model1:** crude model with no covariate adjusted; **Model2:** adjusted for age, gender, race or ethnicity, education level, marriage status and poverty-income ratio; **Model3:** on the basis of model2, further adjusted for smoking status, alcohol drinking, daily total energy intake, physical activity, history of cancer.

***Abbreviations:*** mVOCs, metabolites of volatile organic compounds; *OR*, odd ratio; 95% CI, 95% confident interval; CEMA, N-Acetyl-S-(2-carboxyethyl)-L-cysteine; 3HPMA, N-Acetyl-S-(3-hydroxypropyl)-L-cysteine; AAMA, N-Acetyl-S-(2-carbamoylethyl)-L-cysteine; CYMA, N-Acetyl-S-(2-cyanoethyl)-L-cysteine; DHBMA, N-Acetyl-S-(3,4-dihydroxybutyl)-L-cysteine; MHBMA3, N-Acetyl-S-(4-hydroxy-2-butenyl)-L-cysteine; HMPMA, N-Acetyl-S-(3-hydroxypropyl-1-methyl)-L-cysteine; ATCA, 2-Aminothiazoline-4-carboxylic acid; AMCC, N-Acetyl-S-(N-methylcarbamoyl)-L-cysteine; PGA, Phenylglyoxylic acid; MA, Mandelic acid; 2HPMA, N-Acetyl-S-(2-hydroxypropyl)-L-cysteine; SBMA, N-Acetyl-S-(benzyl)-L-cysteine; 2MHA, 2-Methylhippuric acid; 3,4-MHA, 3- and 4-Methylhippuric acid.

**Table.S3** Association between urine mVOCs and **elevated TG**

| **Urinary mVOCs** | Model 1 | | | Model 2 | | | Model 3 | | |
| --- | --- | --- | --- | --- | --- | --- | --- | --- | --- |
|  | ***OR*** | 95% ***CI*** | *P* value | ***OR*** | 95% ***CI*** | ***P*** value | ***OR*** | 95% ***CI*** | *P* value |
| **CEMA** | —— | —— | —— | —— | —— | —— | —— | —— | —— |
| Q2 | 1.50 | 1.20, 1.87 | **<0.01** | 1.48 | 1.18, 1.86 | **<0.01** | 1.47 | 1.16, 1.85 | **<0.01** |
| Q3 | 1.53 | 1.19, 1.96 | **<0.01** | 1.48 | 1.14, 1.92 | **<0.01** | 1.45 | 1.13, 1.87 | **<0.01** |
| Q4 | 1.72 | 1.38, 2.16 | **<0.01** | 1.69 | 1.33, 2.16 | **<0.01** | 1.65 | 1.29, 2.09 | **<0.01** |
| *P*_trend_ | **<0.01** | | | **<0.01** | | | **<0.01** | | |
| **3HPMA** | —— | —— | —— | —— | —— | —— | —— | —— | —— |
| Q2 | 1.30 | 1.00, 1.69 | **<0.05** | 1.21 | 0.92, 1.59 | 0.20 | 1.21 | 0.92, 1.60 | 0.20 |
| Q3 | 1.56 | 1.25, 1.93 | **<0.01** | 1.47 | 1.17, 1.84 | **<0.01** | 1.49 | 1.19, 1.86 | **<0.01** |
| Q4 | 1.68 | 1.28, 2.20 | **<0.01** | 1.59 | 1.19, 2.13 | **<0.01** | 1.57 | 1.16, 2.12 | **<0.01** |
| *P*_trend_ | **<0.01** | | | **<0.01** | | | **<0.01** | | |
| **AAMA** | —— | —— | —— | —— | —— | —— | —— | —— | —— |
| Q2 | 0.93 | 0.72, 1.20 | 0.60 | 0.9 | 0.69, 1.17 | 0.40 | 0.88 | 0.67, 1.15 | 0.30 |
| Q3 | 0.94 | 0.73, 1.20 | 0.60 | 0.95 | 0.73, 1.23 | 0.70 | 0.91 | 0.71, 1.19 | 0.50 |
| Q4 | 0.89 | 0.69, 1.13 | 0.30 | 0.84 | 0.66, 1.08 | 0.20 | 0.78 | 0.61, 1.00 | 0.05 |
| *P*_trend_ | 0.30 | | | 0.20 | | | 0.07 | | |
| **CYMA** | —— | —— | —— | —— | —— | —— | —— | —— | —— |
| Q2 | 0.83 | 0.66, 1.04 | 0.10 | 0.83 | 0.67, 1.04 | 0.11 | 0.85 | 0.68, 1.06 | 0.15 |
| Q3 | 0.93 | 0.73, 1.18 | 0.50 | 1.01 | 0.79, 1.28 | >0.90 | 1.02 | 0.80, 1.30 | 0.90 |
| Q4 | 1.04 | 0.84, 1.30 | 0.70 | 1.05 | 0.81, 1.35 | 0.70 | 1.04 | 0.76, 1.42 | 0.80 |
| *P*_trend_ | 0.50 | | | 0.40 | | | 0.60 | | |
| **DHBMA** | —— | —— | —— | —— | —— | —— | —— | —— | —— |
| Q2 | 1.24 | 0.98, 1.56 | 0.07 | 1.23 | 0.97, 1.56 | 0.09 | 1.22 | 0.97, 1.55 | 0.09 |
| Q3 | 1.21 | 0.97, 1.51 | 0.1 | 1.18 | 0.94, 1.48 | 0.2 | 1.19 | 0.95, 1.50 | 0.13 |
| Q4 | 1.53 | 1.21, 1.94 | **<0.01** | 1.44 | 1.13, 1.85 | **<0.01** | 1.43 | 1.12, 1.82 | **<0.01** |
| *P*_trend_ | **<0.01** | | | **<0.01** | | | **<0.01** | | |
| **MHBMA3** | —— | —— | —— | —— | —— | —— | —— | —— | —— |
| Q2 | 1.55 | 1.21, 1.98 | **<0.01** | 1.56 | 1.21, 2.02 | **<0.01** | 1.57 | 1.22, 2.03 | **<0.01** |
| Q3 | 1.76 | 1.36, 2.28 | **<0.01** | 1.82 | 1.38, 2.41 | **<0.01** | 1.85 | 1.39, 2.47 | **<0.01** |
| Q4 | 1.78 | 1.38, 2.29 | **<0.01** | 1.76 | 1.34, 2.32 | **<0.01** | 1.76 | 1.31, 2.37 | **<0.01** |
| *P*_trend_ | **<0.01** | | | **<0.01** | | | **<0.01** | | |
| **HMPMA** | —— | —— | —— | —— | —— | —— | —— | —— | —— |
| Q2 | 1.27 | 1.00, 1.62 | 0.05 | 1.28 | 1.00, 1.65 | 0.05 | 1.30 | 1.02, 1.67 | **<0.05** |
| Q3 | 1.81 | 1.44, 2.29 | **<0.01** | 1.85 | 1.43, 2.41 | **<0.01** | 1.90 | 1.46, 2.47 | **<0.01** |
| Q4 | 1.81 | 1.43, 2.28 | **<0.01** | 1.79 | 1.37, 2.35 | **<0.01** | 1.82 | 1.38, 2.40 | **<0.01** |
| *P*_trend_ | **<0.01** | | | **<0.01** | | | **<0.01** | | |
| **ATCA** | —— | —— | —— | —— | —— | —— | —— | —— | —— |
| Q2 | 0.84 | 0.68, 1.04 | 0.11 | 0.90 | 0.71, 1.15 | 0.40 | 0.92 | 0.73, 1.17 | 0.50 |
| Q3 | 0.76 | 0.61, 0.94 | **<0.05** | 0.89 | 0.67, 1.17 | 0.40 | 0.88 | 0.67, 1.16 | 0.40 |
| Q4 | 0.84 | 0.64, 1.10 | 0.20 | 1.08 | 0.77, 1.51 | 0.70 | 1.08 | 0.77, 1.51 | 0.60 |
| *P*_trend_ | 0.20 | | | 0.70 | | | 0.70 | | |
| **AMCC** | —— | —— | —— | —— | —— | —— | —— | —— | —— |
| Q2 | 1.03 | 0.81, 1.32 | 0.80 | 0.89 | 0.69, 1.15 | 0.40 | 0.86 | 0.66, 1.11 | 0.20 |
| Q3 | 0.99 | 0.76, 1.29 | >0.90 | 0.82 | 0.63, 1.07 | 0.14 | 0.77 | 0.58, 1.01 | 0.06 |
| Q4 | 0.97 | 0.71, 1.32 | 0.80 | 0.77 | 0.55, 1.06 | 0.10 | 0.68 | 0.47, 0.98 | **<0.05** |
| *P*_trend_ | 0.80 | | | 0.09 | | | **<0.05** | | |
| **PGA** | —— | —— | —— | —— | —— | —— | —— | —— | —— |
| Q2 | 0.71 | 0.53, 0.96 | **<0.05** | 0.68 | 0.50, 0.93 | **<0.05** | 0.69 | 0.51, 0.94 | **<0.05** |
| Q3 | 0.82 | 0.64, 1.05 | 0.12 | 0.75 | 0.57, 0.98 | **<0.05** | 0.75 | 0.57, 0.98 | **<0.05** |
| Q4 | 0.86 | 0.63, 1.16 | 0.30 | 0.77 | 0.58, 1.03 | 0.08 | 0.76 | 0.58, 1.01 | 0.06 |
| *P*_trend_ | 0.50 | | | 0.14 | | | 0.10 | | |
| **MA** | —— | —— | —— | —— | —— | —— | —— | —— | —— |
| Q2 | 0.97 | 0.72, 1.29 | 0.80 | 0.97 | 0.72, 1.30 | 0.80 | 0.98 | 0.73, 1.33 | >0.90 |
| Q3 | 1.12 | 0.84, 1.48 | 0.40 | 1.14 | 0.87, 1.51 | 0.30 | 1.14 | 0.86, 1.51 | 0.40 |
| Q4 | 1.30 | 1.00, 1.69 | 0.05 | 1.31 | 1.00, 1.72 | 0.05 | 1.31 | 0.99, 1.73 | 0.06 |
| *P*_trend_ | **<0.05** | | | **0.03** | | | **<0.05** | | |
| **2HPMA** | —— | —— | —— | —— | —— | —— | —— | —— | —— |
| Q2 | 0.95 | 0.75, 1.21 | 0.70 | 0.94 | 0.73, 1.22 | 0.70 | 0.93 | 0.72, 1.19 | 0.60 |
| Q3 | 0.88 | 0.67, 1.16 | 0.40 | 0.85 | 0.64, 1.12 | 0.20 | 0.83 | 0.62, 1.09 | 0.20 |
| Q4 | 0.88 | 0.66, 1.17 | 0.40 | 0.88 | 0.64, 1.20 | 0.40 | 0.82 | 0.60, 1.13 | 0.20 |
| *P*_trend_ | 0.30 | | | 0.30 | | | 0.20 | | |
| **SBMA** | —— | —— | —— | —— | —— | —— | —— | —— | —— |
| Q2 | 0.86 | 0.65, 1.12 | 0.30 | 0.91 | 0.67, 1.24 | 0.60 | 0.91 | 0.67, 1.24 | 0.60 |
| Q3 | 0.84 | 0.67, 1.05 | 0.13 | 0.9 | 0.70, 1.15 | 0.40 | 0.90 | 0.70, 1.16 | 0.40 |
| Q4 | 0.73 | 0.57, 0.92 | **<0.01** | 0.81 | 0.62, 1.06 | 0.12 | 0.81 | 0.62, 1.07 | 0.14 |
| *P*_trend_ | **<0.01** | | | 0.11 | | | 0.12 | | |
| **2MHA** | —— | —— | —— | —— | —— | —— | —— | —— | —— |
| Q2 | 1.18 | 0.92, 1.50 | 0.20 | 1.14 | 0.87, 1.49 | 0.30 | 1.16 | 0.89, 1.52 | 0.30 |
| Q3 | 1.07 | 0.83, 1.38 | 0.60 | 1.03 | 0.80, 1.33 | 0.80 | 1.02 | 0.79, 1.32 | 0.90 |
| Q4 | 1.07 | 0.81, 1.41 | 0.60 | 0.97 | 0.74, 1.29 | 0.90 | 0.95 | 0.72, 1.25 | 0.70 |
| *P*_trend_ | 0.80 | | | 0.70 | | | 0.50 | | |
| **3,4MHA** | —— | —— | —— | —— | —— | —— | —— | —— | —— |
| Q2 | 1.21 | 0.97, 1.51 | 0.06 | 1.16 | 0.91, 1.47 | 0.20 | 1.19 | 0.94, 1.51 | 0.15 |
| Q3 | 1.28 | 1.03, 1.59 | **<0.05** | 1.25 | 0.97, 1.60 | 0.09 | 1.26 | 0.97, 1.63 | 0.08 |
| Q4 | 0.99 | 0.72, 1.36 | >0.90 | 0.90 | 0.66, 1.24 | 0.50 | 0.87 | 0.63, 1.21 | 0.40 |
| *P*_trend_ | >0.90 | | | 0.60 | | | 0.50 | | |

***Notes:*** Urinary mVOCs were introduced into logistic regression models as categorical variables. All results were presented as OR, 95% *CI*, *P* value, and *P*_trend_. **Model1:** crude model with no covariate adjusted; **Model2:** adjusted for age, gender, race or ethnicity, education level, marriage status and poverty-income ratio; **Model3:** on the basis of model2, further adjusted for smoking status, alcohol drinking, daily total energy intake, physical activity, history of cancer.

***Abbreviations:*** mVOCs, metabolites of volatile organic compounds; TG, triglyceride; *OR*, odd ratio; 95% CI, 95% confident interval; CEMA, N-Acetyl-S-(2-carboxyethyl)-L-cysteine; 3HPMA, N-Acetyl-S-(3-hydroxypropyl)-L-cysteine; AAMA, N-Acetyl-S-(2-carbamoylethyl)-L-cysteine; CYMA, N-Acetyl-S-(2-cyanoethyl)-L-cysteine; DHBMA, N-Acetyl-S-(3,4-dihydroxybutyl)-L-cysteine; MHBMA3, N-Acetyl-S-(4-hydroxy-2-butenyl)-L-cysteine; HMPMA, N-Acetyl-S-(3-hydroxypropyl-1-methyl)-L-cysteine; ATCA, 2-Aminothiazoline-4-carboxylic acid; AMCC, N-Acetyl-S-(N-methylcarbamoyl)-L-cysteine; PGA, Phenylglyoxylic acid; MA, Mandelic acid; 2HPMA, N-Acetyl-S-(2-hydroxypropyl)-L-cysteine; SBMA, N-Acetyl-S-(benzyl)-L-cysteine; 2MHA, 2-Methylhippuric acid; 3,4-MHA, 3- and 4-Methylhippuric acid.

**Table.S4** Association between urine mVOCs and **reduced HDL**

| **Urinary mVOCs** | Model 1 | | | Model 2 | | | Model 3 | | |
| --- | --- | --- | --- | --- | --- | --- | --- | --- | --- |
|  | ***OR*** | 95% CI | *P* value | ***OR*** | 95% CI | *P* value | ***OR*** | 95% CI | *P* value |
| **CEMA** | —— | —— | —— | —— | —— | —— | —— | —— | —— |
| Q2 | 1.10 | 0.86, 1.41 | 0.40 | 1.15 | 0.90, 1.46 | 0.30 | 1.13 | 0.89, 1.43 | 0.30 |
| Q3 | 1.27 | 1.06, 1.51 | **<0.05** | 1.24 | 1.03, 1.49 | **<0.05** | 1.23 | 1.02, 1.49 | **<0.05** |
| Q4 | 1.49 | 1.19, 1.87 | **<0.01** | 1.42 | 1.15, 1.76 | **<0.01** | 1.41 | 1.14, 1.75 | **<0.01** |
| *P*_trend_ | **<0.01** | | | **<0.01** | | | **<0.01** | | |
| **3HPMA** | —— | —— | —— | —— | —— | —— | —— | —— | —— |
| Q2 | 1.07 | 0.82, 1.38 | 0.60 | 1.10 | 0.84, 1.44 | 0.50 | 1.12 | 0.86, 1.46 | 0.40 |
| Q3 | 1.21 | 0.96, 1.52 | 0.11 | 1.23 | 0.97, 1.58 | 0.09 | 1.27 | 0.99, 1.63 | 0.06 |
| Q4 | 1.38 | 1.08, 1.78 | **<0.05** | 1.22 | 0.94, 1.57 | 0.13 | 1.21 | 0.91, 1.61 | 0.20 |
| *P*_trend_ | **<0.05** | | | 0.08 | | | 0.12 | | |
| **AAMA** | —— | —— | —— | —— | —— | —— | —— | —— | —— |
| Q2 | 0.97 | 0.76, 1.26 | 0.80 | 0.98 | 0.76, 1.26 | 0.90 | 0.97 | 0.75, 1.26 | 0.80 |
| Q3 | 0.89 | 0.70, 1.13 | 0.30 | 0.88 | 0.68, 1.13 | 0.30 | 0.86 | 0.67, 1.12 | 0.30 |
| Q4 | 0.95 | 0.77, 1.18 | 0.60 | 0.81 | 0.66, 0.99 | **<0.05** | 0.76 | 0.62, 0.94 | **<0.05** |
| *P*_trend_ | 0.50 | | | **<0.05** | | | **<0.05** | | |
| **CYMA** | —— | —— | —— | —— | —— | —— | —— | —— | —— |
| Q2 | 0.82 | 0.64, 1.04 | 0.09 | 0.85 | 0.67, 1.07 | 0.20 | 0.86 | 0.69, 1.08 | 0.20 |
| Q3 | 0.75 | 0.59, 0.96 | **<0.05** | 0.73 | 0.58, 0.93 | **<0.05** | 0.76 | 0.60, 0.96 | **<0.05** |
| Q4 | 1.20 | 0.95, 1.53 | 0.13 | 0.98 | 0.77, 1.26 | 0.90 | 1.04 | 0.79, 1.38 | 0.80 |
| *P*_trend_ | 0.20 | | | 0.60 | | | >0.90 | | |
| **DHBMA** | —— | —— | —— | —— | —— | —— | —— | —— | —— |
| Q2 | 0.98 | 0.76, 1.28 | >0.90 | 0.97 | 0.75, 1.26 | 0.80 | 0.97 | 0.75, 1.26 | 0.80 |
| Q3 | 0.91 | 0.71, 1.17 | 0.50 | 0.91 | 0.71, 1.17 | 0.50 | 0.91 | 0.71, 1.17 | 0.50 |
| Q4 | 1.03 | 0.79, 1.34 | 0.80 | 0.98 | 0.75, 1.29 | 0.90 | 0.98 | 0.75, 1.29 | 0.90 |
| *P*_trend_ | >0.90 | | | 0.80 | | | 0.90 | | |
| **MHBMA3** | —— | —— | —— | —— | —— | —— | —— | —— | —— |
| Q2 | 0.97 | 0.77, 1.23 | 0.80 | 1.04 | 0.81, 1.33 | 0.80 | 1.04 | 0.82, 1.33 | 0.70 |
| Q3 | 1.11 | 0.90, 1.37 | 0.30 | 1.16 | 0.94, 1.44 | 0.20 | 1.19 | 0.97, 1.47 | 0.10 |
| Q4 | 1.40 | 1.10, 1.79 | **<0.05** | 1.21 | 0.96, 1.53 | 0.10 | 1.23 | 0.96, 1.58 | 0.11 |
| *P*_trend_ | **<0.05** | | | 0.07 | | | 0.06 | | |
| **HMPMA** | —— | —— | —— | —— | —— | —— | —— | —— | —— |
| Q2 | 0.73 | 0.56, 0.95 | **<0.05** | 0.77 | 0.60, 1.00 | **0.05** | 0.79 | 0.61, 1.02 | 0.07 |
| Q3 | 0.89 | 0.69, 1.15 | 0.40 | 0.97 | 0.75, 1.25 | 0.80 | 1.00 | 0.78, 1.29 | >0.90 |
| Q4 | 1.32 | 1.03, 1.68 | **<0.05** | 1.19 | 0.94, 1.52 | 0.15 | 1.22 | 0.95, 1.58 | 0.12 |
| *P*_trend_ | **<0.05** | | | 0.05 | | | **<0.05** | | |
| **ATCA** | —— | —— | —— | —— | —— | —— | —— | —— | —— |
| Q2 | 0.89 | 0.68, 1.18 | 0.40 | 0.79 | 0.60, 1.02 | 0.07 | 0.82 | 0.63, 1.07 | 0.15 |
| Q3 | 0.93 | 0.73, 1.17 | 0.50 | 0.73 | 0.57, 0.92 | **<0.05** | 0.73 | 0.58, 0.93 | **<0.05** |
| Q4 | 1.01 | 0.81, 1.26 | >0.90 | 0.71 | 0.55, 0.91 | **<0.05** | 0.73 | 0.57, 0.93 | **<0.05** |
| *P*_trend_ | 0.80 | | | **<0.05** | | | **<0.05** | | |
| **AMCC** | —— | —— | —— | —— | —— | —— | —— | —— | —— |
| Q2 | 0.84 | 0.66, 1.06 | 0.14 | 0.81 | 0.62, 1.05 | 0.11 | 0.79 | 0.61, 1.03 | 0.08 |
| Q3 | 0.80 | 0.60, 1.06 | 0.11 | 0.77 | 0.58, 1.01 | 0.06 | 0.75 | 0.57, 0.99 | **<0.05** |
| Q4 | 1.01 | 0.80, 1.28 | >0.90 | 0.86 | 0.67, 1.11 | 0.20 | 0.82 | 0.62, 1.09 | 0.20 |
| *P*_trend_ | >0.90 | | | >0.90 | | | 0.20 | | |
| **PGA** | —— | —— | —— | —— | —— | —— | —— | —— | —— |
| Q2 | 0.85 | 0.67, 1.09 | 0.20 | 0.88 | 0.69, 1.12 | 0.30 | 0.90 | 0.71, 1.13 | 0.40 |
| Q3 | 0.72 | 0.58, 0.90 | **<0.05** | 0.72 | 0.57, 0.91 | **<0.05** | 0.72 | 0.58, 0.91 | **<0.05** |
| Q4 | 0.85 | 0.66, 1.11 | 0.20 | 0.76 | 0.58, 1.00 | **0.05** | 0.77 | 0.59, 1.00 | **0.05** |
| *P*_trend_ | 0.11 | | | **<0.05** | | | **<0.05** | | |
| **MA** | —— | —— | —— | —— | —— | —— | —— | —— | —— |
| Q2 | 0.81 | 0.62, 1.04 | 0.10 | 0.78 | 0.60, 1.02 | 0.07 | 0.80 | 0.61, 1.05 | 0.11 |
| Q3 | 0.87 | 0.66, 1.13 | 0.30 | 0.81 | 0.62, 1.05 | 0.11 | 0.81 | 0.62, 1.06 | 0.12 |
| Q4 | 1.14 | 0.93, 1.39 | 0.20 | 0.96 | 0.77, 1.19 | 0.70 | 0.98 | 0.78, 1.22 | 0.80 |
| *P*_trend_ | 0.13 | | | 0.80 | | | 0.90 | | |
| **2HPMA** | —— | —— | —— | —— | —— | —— | —— | —— | —— |
| Q2 | 1.13 | 0.88, 1.44 | 0.30 | 1.10 | 0.85, 1.42 | 0.40 | 1.10 | 0.86, 1.42 | 0.40 |
| Q3 | 1.02 | 0.80, 1.30 | 0.90 | 0.99 | 0.78, 1.27 | >0.90 | 1.00 | 0.79, 1.26 | >0.90 |
| Q4 | 1.11 | 0.89, 1.39 | 0.30 | 1.03 | 0.83, 1.28 | 0.80 | 0.99 | 0.79, 1.24 | >0.90 |
| *P*_trend_ | 0.60 | | | >0.90 | | | 0.80 | | |
| **SBMA** | —— | —— | —— | —— | —— | —— | —— | —— | —— |
| Q2 | 0.84 | 0.66, 1.06 | 0.14 | 0.85 | 0.66, 1.10 | 0.20 | 0.86 | 0.66, 1.12 | 0.30 |
| Q3 | 0.77 | 0.65, 0.91 | **<0.01** | 0.75 | 0.64, 0.87 | **<0.01** | 0.75 | 0.64, 0.89 | **<0.01** |
| Q4 | 0.74 | 0.57, 0.97 | **<0.05** | 0.73 | 0.56, 0.96 | **<0.05** | 0.76 | 0.59, 0.99 | **<0.05** |
| *P*_trend_ | **<0.05** | | | **<0.05** | | | **<0.05** | | |
| **2MHA** | —— | —— | —— | —— | —— | —— | —— | —— | —— |
| Q2 | 0.94 | 0.72, 1.22 | 0.60 | 0.90 | 0.69, 1.17 | 0.40 | 0.92 | 0.70, 1.20 | 0.50 |
| Q3 | 1.01 | 0.76, 1.32 | >0.90 | 0.95 | 0.73, 1.24 | 0.70 | 0.96 | 0.73, 1.26 | 0.80 |
| Q4 | 0.98 | 0.79, 1.22 | 0.90 | 0.84 | 0.68, 1.03 | 0.10 | 0.84 | 0.67, 1.06 | 0.14 |
| *P*_trend_ | >0.90 | | | 0.20 | | | 0.20 | | |
| **3,4MHA** | —— | —— | —— | —— | —— | —— | —— | —— | —— |
| Q2 | 0.92 | 0.68, 1.23 | 0.60 | 0.90 | 0.68, 1.21 | 0.50 | 0.93 | 0.70, 1.24 | 0.60 |
| Q3 | 1.03 | 0.80, 1.33 | 0.80 | 1.03 | 0.80, 1.31 | 0.80 | 1.05 | 0.82, 1.35 | 0.70 |
| Q4 | 1.06 | 0.82, 1.35 | 0.70 | 0.91 | 0.72, 1.16 | 0.40 | 0.91 | 0.70, 1.19 | 0.50 |
| *P*_trend_ | 0.40 | | | 0.70 | | | 0.70 | | |

***Notes:*** Urinary mVOCs were introduced into logistic regression models as categorical variables. All results were presented as OR, 95% *CI*, *P* value, and *P*_trend_. **Model1:** crude model with no covariate adjusted; **Model2:** adjusted for age, gender, race or ethnicity, education level, marriage status and poverty-income ratio; **Model3:** on the basis of model2, further adjusted for smoking status, alcohol drinking, daily total energy intake, physical activity, history of cancer.

***Abbreviations:*** mVOCs, metabolites of volatile organic compounds; HDL, high-density lipoprotein; *OR*, odd ratio; 95% CI, 95% confident interval; CEMA, N-Acetyl-S-(2-carboxyethyl)-L-cysteine; 3HPMA, N-Acetyl-S-(3-hydroxypropyl)-L-cysteine; AAMA, N-Acetyl-S-(2-carbamoylethyl)-L-cysteine; CYMA, N-Acetyl-S-(2-cyanoethyl)-L-cysteine; DHBMA, N-Acetyl-S-(3,4-dihydroxybutyl)-L-cysteine; MHBMA3, N-Acetyl-S-(4-hydroxy-2-butenyl)-L-cysteine; HMPMA, N-Acetyl-S-(3-hydroxypropyl-1-methyl)-L-cysteine; ATCA, 2-Aminothiazoline-4-carboxylic acid; AMCC, N-Acetyl-S-(N-methylcarbamoyl)-L-cysteine; PGA, Phenylglyoxylic acid; MA, Mandelic acid; 2HPMA, N-Acetyl-S-(2-hydroxypropyl)-L-cysteine; SBMA, N-Acetyl-S-(benzyl)-L-cysteine; 2MHA, 2-Methylhippuric acid; 3,4-MHA, 3- and 4-Methylhippuric acid.

**Table.S5** Association between urine mVOCs and **high BP**

| **Urinary mVOCs** | Model 1 | | | Model 2 | | | Model 3 | | |
| --- | --- | --- | --- | --- | --- | --- | --- | --- | --- |
|  | ***OR*** | 95% CI | *P* value | ***OR*** | 95% CI | *P* value | ***OR*** | 95% CI | *P* value |
| **CEMA** | —— | —— | —— | —— | —— | —— | —— | —— | —— |
| Q2 | 1.40 | 1.15, 1.71 | **<0.01** | 1.19 | 0.94, 1.51 | 0.14 | 1.19 | 0.93, 1.51 | 0.20 |
| Q3 | 1.82 | 1.42, 2.33 | **<0.01** | 1.40 | 1.04, 1.89 | **<0.05** | 1.40 | 1.03, 1.88 | **<0.05** |
| Q4 | 2.00 | 1.62, 2.49 | **<0.01** | 1.48 | 1.17, 1.88 | **<0.01** | 1.49 | 1.15, 1.94 | **<0.01** |
| *P*_trend_ | **<0.01** | | | **<0.01** | | | **<0.01** | | |
| **3HPMA** | —— | —— | —— | —— | —— | —— | —— | —— | —— |
| Q2 | 1.05 | 0.82, 1.34 | 0.70 | 1.04 | 0.77, 1.40 | 0.80 | 1.05 | 0.79, 1.40 | 0.70 |
| Q3 | 1.18 | 0.96, 1.45 | 0.12 | 1.18 | 0.90, 1.54 | 0.20 | 1.18 | 0.90, 1.55 | 0.20 |
| Q4 | 1.07 | 0.85, 1.35 | 0.60 | 1.16 | 0.87, 1.54 | 0.30 | 1.14 | 0.82, 1.58 | 0.40 |
| *P*_trend_ | 0.30 | | | 0.20 | | | 0.30 | | |
| **AAMA** | —— | —— | —— | —— | —— | —— | —— | —— | —— |
| Q2 | 1.10 | 0.91, 1.33 | 0.30 | 1.09 | 0.87, 1.37 | 0.40 | 1.09 | 0.86, 1.37 | 0.50 |
| Q3 | 0.98 | 0.79, 1.22 | 0.90 | 0.99 | 0.77, 1.27 | >0.90 | 0.97 | 0.74, 1.26 | 0.80 |
| Q4 | 0.99 | 0.79, 1.24 | >0.90 | 1.04 | 0.80, 1.36 | 0.80 | 1.01 | 0.76, 1.35 | >0.90 |
| *P*_trend_ | 0.70 | | | >0.90 | | | 0.90 | | |
| **CYMA** | —— | —— | —— | —— | —— | —— | —— | —— | —— |
| Q2 | 1.01 | 0.84, 1.20 | >0.90 | 0.95 | 0.75, 1.19 | 0.60 | 0.96 | 0.76, 1.21 | 0.70 |
| Q3 | 0.97 | 0.79, 1.19 | 0.80 | 0.97 | 0.77, 1.22 | 0.80 | 0.95 | 0.75, 1.20 | 0.70 |
| Q4 | 0.93 | 0.74, 1.18 | 0.50 | 1.07 | 0.82, 1.39 | 0.60 | 1.04 | 0.76, 1.44 | 0.80 |
| *P*_trend_ | 0.50 | | | 0.60 | | | 0.80 | | |
| **DHBMA** | —— | —— | —— | —— | —— | —— | —— | —— | —— |
| Q2 | 1.28 | 1.02, 1.60 | **<0.05** | 1.06 | 0.84, 1.35 | 0.60 | 1.06 | 0.83, 1.35 | 0.60 |
| Q3 | 1.64 | 1.30, 2.06 | **<0.01** | 1.22 | 0.95, 1.56 | 0.12 | 1.26 | 0.97, 1.64 | 0.08 |
| Q4 | 2.27 | 1.86, 2.78 | **<0.01** | 1.33 | 1.05, 1.70 | **<0.05** | 1.36 | 1.04, 1.77 | **<0.05** |
| *P*_trend_ | **<0.01** | | | **<0.05** | | | **<0.05** | | |
| **MHBMA3** | —— | —— | —— | —— | —— | —— | —— | —— | —— |
| Q2 | 1.27 | 1.02, 1.59 | **<0.05** | 0.92 | 0.71, 1.19 | 0.50 | 0.93 | 0.72, 1.20 | 0.60 |
| Q3 | 1.14 | 0.93, 1.40 | 0.20 | 0.79 | 0.60, 1.04 | 0.09 | 0.81 | 0.61, 1.06 | 0.12 |
| Q4 | 1.23 | 1.01, 1.50 | **<0.05** | 1.04 | 0.81, 1.33 | 0.80 | 1.02 | 0.76, 1.38 | 0.90 |
| *P*_trend_ | 0.11 | | | >0.9 | | | 0.90 | | |
| **HMPMA** | —— | —— | —— | —— | —— | —— | —— | —— | —— |
| Q2 | 1.48 | 1.19, 1.85 | **<0.01** | 1.09 | 0.81, 1.46 | 0.60 | 1.08 | 0.80, 1.46 | 0.60 |
| Q3 | 2.15 | 1.74, 2.67 | **<0.01** | 1.58 | 1.24, 2.00 | **<0.01** | 1.56 | 1.23, 1.97 | **<0.01** |
| Q4 | 1.50 | 1.25, 1.80 | **<0.01** | 1.12 | 0.90, 1.39 | 0.30 | 1.08 | 0.84, 1.38 | 0.50 |
| *P*_trend_ | **<0.01** | | | 0.05 | | | 0.13 | | |
| **ATCA** | —— | —— | —— | —— | —— | —— | —— | —— | —— |
| Q2 | 0.95 | 0.75, 1.20 | 0.70 | 0.96 | 0.74, 1.26 | 0.80 | 0.97 | 0.74, 1.27 | 0.80 |
| Q3 | 0.82 | 0.66, 1.01 | 0.07 | 0.85 | 0.64, 1.12 | 0.20 | 0.84 | 0.63, 1.12 | 0.20 |
| Q4 | 0.98 | 0.81, 1.18 | 0.80 | 1.00 | 0.73, 1.37 | >0.9 | 0.99 | 0.71, 1.36 | >0.9 |
| *P*_trend_ | 0.50 | | | 0.80 | | | 0.70 | | |
| **AMCC** | —— | —— | —— | —— | —— | —— | —— | —— | —— |
| Q2 | 1.16 | 0.91, 1.48 | 0.20 | 0.92 | 0.66, 1.28 | 0.60 | 0.89 | 0.64, 1.23 | 0.50 |
| Q3 | 1.26 | 0.99, 1.61 | **<0.05** | 0.87 | 0.66, 1.16 | 0.30 | 0.82 | 0.62, 1.09 | 0.20 |
| Q4 | 1.40 | 1.11, 1.77 | **<0.05** | 0.91 | 0.66, 1.25 | 0.50 | 0.85 | 0.60, 1.21 | 0.40 |
| *P*_trend_ | <0.01 | | | 0.50 | | | 0.30 | | |
| **PGA** | —— | —— | —— | —— | —— | —— | —— | —— | —— |
| Q2 | 1.19 | 0.97, 1.45 | 0.09 | 0.99 | 0.77, 1.27 | >0.90 | 1.02 | 0.79, 1.31 | >0.90 |
| Q3 | 1.31 | 1.05, 1.64 | **<0.05** | 0.89 | 0.68, 1.15 | 0.30 | 0.91 | 0.69, 1.19 | 0.50 |
| Q4 | 1.42 | 1.10, 1.83 | **<0.05** | 0.93 | 0.70, 1.24 | 0.60 | 0.93 | 0.70, 1.24 | 0.60 |
| *P*_trend_ | **<0.05** | | | 0.50 | | | 0.50 | | |
| **MA** | —— | —— | —— | —— | —— | —— | —— | —— | —— |
| Q2 | 0.84 | 0.67, 1.04 | 0.10 | 0.82 | 0.62, 1.08 | 0.20 | 0.81 | 0.61, 1.06 | 0.12 |
| Q3 | 0.97 | 0.79, 1.20 | 0.80 | 0.96 | 0.73, 1.27 | 0.80 | 0.95 | 0.72, 1.25 | 0.70 |
| Q4 | 1.07 | 0.87, 1.31 | 0.50 | 1.08 | 0.86, 1.37 | 0.50 | 1.07 | 0.84, 1.37 | 0.60 |
| *P*_trend_ | 0.30 | | | 0.30 | | | 0.40 | | |
| **2HPMA** | —— | —— | —— | —— | —— | —— | —— | —— | —— |
| Q2 | 1.15 | 0.95, 1.41 | 0.20 | 1.15 | 0.90, 1.46 | 0.30 | 1.14 | 0.89, 1.46 | 0.30 |
| Q3 | 1.00 | 0.82, 1.22 | >0.90 | 1.01 | 0.80, 1.27 | >0.90 | 0.99 | 0.78, 1.26 | >0.90 |
| Q4 | 0.90 | 0.74, 1.10 | 0.30 | 0.88 | 0.70, 1.11 | 0.30 | 0.85 | 0.66, 1.08 | 0.20 |
| *P*_trend_ | 0.20 | | | 0.20 | | | 0.10 | | |
| **SBMA** | —— | —— | —— | —— | —— | —— | —— | —— | —— |
| Q2 | 1.07 | 0.86, 1.32 | 0.50 | 0.97 | 0.75, 1.25 | 0.80 | 0.97 | 0.74, 1.26 | 0.80 |
| Q3 | 1.15 | 0.95, 1.39 | 0.14 | 0.91 | 0.72, 1.13 | 0.40 | 0.91 | 0.73, 1.14 | 0.40 |
| Q4 | 1.16 | 0.93, 1.44 | 0.20 | 0.86 | 0.67, 1.10 | 0.20 | 0.87 | 0.69, 1.10 | 0.20 |
| *P*_trend_ | 0.14 | | | 0.20 | | | 0.20 | | |
| **2MHA** | —— | —— | —— | —— | —— | —— | —— | —— | —— |
| Q2 | 0.87 | 0.71, 1.07 | 0.20 | 0.87 | 0.67, 1.13 | 0.30 | 0.86 | 0.67, 1.12 | 0.30 |
| Q3 | 0.89 | 0.74, 1.07 | 0.20 | 0.90 | 0.69, 1.16 | 0.40 | 0.89 | 0.68, 1.16 | 0.40 |
| Q4 | 0.87 | 0.70, 1.09 | 0.20 | 0.80 | 0.61, 1.06 | 0.12 | 0.78 | 0.58, 1.05 | 0.10 |
| *P*_trend_ | 0.20 | | | 0.14 | | | 0.12 | | |
| **3,4MHA** | —— | —— | —— | —— | —— | —— | —— | —— | —— |
| Q2 | 1.12 | 0.87, 1.44 | 0.40 | 0.96 | 0.71, 1.28 | 0.80 | 0.98 | 0.73, 1.33 | >0.9 |
| Q3 | 1.12 | 0.92, 1.38 | 0.30 | 0.93 | 0.69, 1.24 | 0.60 | 0.94 | 0.70, 1.26 | 0.70 |
| Q4 | 1.10 | 0.88, 1.39 | 0.40 | 0.92 | 0.69, 1.22 | 0.60 | 0.92 | 0.69, 1.23 | 0.60 |
| *P*_trend_ | 0.40 | | | 0.50 | | | 0.50 | | |

***Notes:*** Urinary mVOCs were introduced into logistic regression models as categorical variables. All results were presented as OR, 95% *CI*, *P* value, and *P*_trend_. **Model1:** crude model with no covariate adjusted; **Model2:** adjusted for age, gender, race or ethnicity, education level, marriage status and poverty-income ratio; **Model3:** on the basis of model2, further adjusted for smoking status, alcohol drinking, daily total energy intake, physical activity, history of cancer.

***Abbreviations:*** mVOCs, metabolites of volatile organic compounds; BP, blood pressure; *OR*, odd ratio; 95% CI, 95% confident interval; CEMA, N-Acetyl-S-(2-carboxyethyl)-L-cysteine; 3HPMA, N-Acetyl-S-(3-hydroxypropyl)-L-cysteine; AAMA, N-Acetyl-S-(2-carbamoylethyl)-L-cysteine; CYMA, N-Acetyl-S-(2-cyanoethyl)-L-cysteine; DHBMA, N-Acetyl-S-(3,4-dihydroxybutyl)-L-cysteine; MHBMA3, N-Acetyl-S-(4-hydroxy-2-butenyl)-L-cysteine; HMPMA, N-Acetyl-S-(3-hydroxypropyl-1-methyl)-L-cysteine; ATCA, 2-Aminothiazoline-4-carboxylic acid; AMCC, N-Acetyl-S-(N-methylcarbamoyl)-L-cysteine; PGA, Phenylglyoxylic acid; MA, Mandelic acid; 2HPMA, N-Acetyl-S-(2-hydroxypropyl)-L-cysteine; SBMA, N-Acetyl-S-(benzyl)-L-cysteine; 2MHA, 2-Methylhippuric acid; 3,4-MHA, 3- and 4-Methylhippuric acid.

**Table.S6** Association between urine mVOCs and impaired **FBG**

| **Urinary mVOCs** | Model 1 | | | Model 2 | | | Model 3 | | |
| --- | --- | --- | --- | --- | --- | --- | --- | --- | --- |
|  | ***OR*** | 95% CI | *P* value | ***OR*** | 95% CI | *P* value | ***OR*** | 95% CI | *P* value |
| **CEMA** | —— | —— | —— | —— | —— | —— | —— | —— | —— |
| Q2 | 1.25 | 0.99, 1.59 | 0.06 | 1.10 | 0.85, 1.42 | 0.50 | 1.09 | 0.83, 1.43 | 0.50 |
| Q3 | 1.80 | 1.39, 2.32 | **<0.01** | 1.43 | 1.09, 1.87 | **<0.05** | 1.45 | 1.09, 1.93 | **<0.05** |
| Q4 | 1.66 | 1.23, 2.26 | **<0.01** | 1.28 | 0.95, 1.73 | 0.11 | 1.31 | 0.96, 1.80 | 0.09 |
| *P*_trend_ | **<0.01** | | | **<0.05** | | | **<0.05** | | |
| **3HPMA** | —— | —— | —— | —— | —— | —— | —— | —— | —— |
| Q2 | 1.11 | 0.80, 1.55 | 0.50 | 1.10 | 0.79, 1.53 | 0.60 | 1.11 | 0.80, 1.55 | 0.50 |
| Q3 | 1.65 | 1.27, 2.13 | **<0.01** | 1.63 | 1.23, 2.15 | **<0.01** | 1.70 | 1.29, 2.23 | **<0.01** |
| Q4 | 1.18 | 0.86, 1.61 | 0.30 | 1.19 | 0.87, 1.63 | 0.30 | 1.25 | 0.91, 1.72 | 0.20 |
| *P*_trend_ | 0.07 | | | 0.06 | | | **<0.05** | | |
| **AAMA** | —— | —— | —— | —— | —— | —— | —— | —— | —— |
| Q2 | 0.89 | 0.65, 1.22 | 0.50 | 0.86 | 0.62, 1.20 | 0.40 | 0.86 | 0.61, 1.21 | 0.40 |
| Q3 | 0.80 | 0.63, 1.03 | 0.08 | 0.79 | 0.62, 1.01 | 0.06 | 0.78 | 0.61, 1.00 | 0.05 |
| Q4 | 0.66 | 0.49, 0.88 | **<0.05** | 0.63 | 0.48, 0.83 | **<0.01** | 0.63 | 0.46, 0.86 | **<0.01** |
| *P*_trend_ | **<0.05** | | | **<0.01** | | | **<0.01** | | |
| **CYMA** | —— | —— | —— | —— | —— | —— | —— | —— | —— |
| Q2 | 1.10 | 0.79, 1.52 | 0.60 | 1.08 | 0.78, 1.51 | 0.60 | 1.10 | 0.79, 1.54 | 0.60 |
| Q3 | 0.93 | 0.71, 1.22 | 0.60 | 0.94 | 0.71, 1.24 | 0.60 | 0.96 | 0.73, 1.27 | 0.80 |
| Q4 | 0.77 | 0.57, 1.05 | 0.10 | 0.78 | 0.57, 1.07 | 0.12 | 0.81 | 0.59, 1.12 | 0.20 |
| *P*_trend_ | 0.06 | | | 0.08 | | | 0.13 | | |
| **DHBMA** | —— | —— | —— | —— | —— | —— | —— | —— | —— |
| Q2 | 0.86 | 0.64, 1.16 | 0.30 | 0.71 | 0.51, 1.00 | 0.05 | 0.72 | 0.51, 1.03 | 0.07 |
| Q3 | 1.20 | 0.86, 1.66 | 0.30 | 0.90 | 0.64, 1.27 | 0.50 | 0.94 | 0.66, 1.33 | 0.70 |
| Q4 | 1.74 | 1.30, 2.35 | **<0.01** | 1.10 | 0.77, 1.57 | 0.60 | 1.17 | 0.79, 1.72 | 0.40 |
| *P*_trend_ | **<0.01** | | | 0.30 | | | 0.20 | | |
| **MHBMA3** | —— | —— | —— | —— | —— | —— | —— | —— | —— |
| Q2 | 1.43 | 1.12, 1.84 | **<0.05** | 1.18 | 0.90, 1.56 | 0.20 | 1.19 | 0.90, 1.58 | 0.20 |
| Q3 | 1.73 | 1.35, 2.22 | **<0.01** | 1.41 | 1.10, 1.82 | **<0.05** | 1.45 | 1.12, 1.87 | **<0.05** |
| Q4 | 1.28 | 0.91, 1.82 | 0.20 | 1.08 | 0.78, 1.51 | 0.60 | 1.14 | 0.80, 1.62 | 0.50 |
| *P*_trend_ | 0.09 | | | 0.40 | | | 0.30 | | |
| **HMPMA** | —— | —— | —— | —— | —— | —— | —— | —— | —— |
| Q2 | 1.69 | 1.24, 2.29 | **<0.01** | 1.39 | 1.01, 1.91 | **0.05** | 1.45 | 1.05, 2.00 | **<0.05** |
| Q3 | 2.49 | 1.83, 3.40 | **<0.01** | 2.01 | 1.45, 2.78 | **<0.01** | 2.14 | 1.56, 2.93 | **<0.01** |
| Q4 | 2.16 | 1.59, 2.94 | **<0.01** | 1.76 | 1.31, 2.37 | **<0.01** | 1.98 | 1.48, 2.64 | **<0.01** |
| *P*_trend_ | **<0.01** | | | **<0.01** | | | **<0.01** | | |
| **ATCA** | —— | —— | —— | —— | —— | —— | —— | —— | —— |
| Q2 | 0.92 | 0.70, 1.22 | 0.60 | 0.94 | 0.70, 1.27 | 0.70 | 0.99 | 0.73, 1.33 | >0.90 |
| Q3 | 1.04 | 0.83, 1.29 | 0.70 | 1.12 | 0.87, 1.45 | 0.40 | 1.16 | 0.91, 1.48 | 0.20 |
| Q4 | 1.27 | 0.96, 1.68 | 0.09 | 1.36 | 0.97, 1.89 | 0.07 | 1.40 | 1.02, 1.93 | **<0.05** |
| *P*_trend_ | **<0.05** | | | **<0.05** | | | **<0.05** | | |
| **AMCC** | —— | —— | —— | —— | —— | —— | —— | —— | —— |
| Q2 | 1.47 | 1.11, 1.95 | 0.01 | 1.27 | 0.92, 1.75 | 0.14 | 1.28 | 0.92, 1.77 | 0.14 |
| Q3 | 1.32 | 0.98, 1.78 | 0.07 | 1.03 | 0.75, 1.40 | 0.90 | 1.04 | 0.76, 1.43 | 0.80 |
| Q4 | 1.43 | 1.09, 1.86 | **<0.05** | 1.05 | 0.79, 1.38 | 0.70 | 1.09 | 0.78, 1.50 | 0.60 |
| *P*_trend_ | **<0.05** | | | 0.90 | | | >0.90 | | |
| **PGA** | —— | —— | —— | —— | —— | —— | —— | —— | —— |
| Q2 | 1.23 | 0.92, 1.64 | 0.20 | 1.10 | 0.81, 1.48 | 0.50 | 1.12 | 0.83, 1.52 | 0.40 |
| Q3 | 1.29 | 1.00, 1.67 | 0.05 | 0.99 | 0.76, 1.29 | >0.9 | 1.01 | 0.78, 1.30 | >0.9 |
| Q4 | 1.30 | 0.95, 1.76 | 0.10 | 0.95 | 0.68, 1.33 | 0.80 | 1.01 | 0.72, 1.40 | >0.9 |
| *P*_trend_ | 0.09 | | | 0.60 | | | 0.90 | | |
| **MA** | —— | —— | —— | —— | —— | —— | —— | —— | —— |
| Q2 | 1.01 | 0.76, 1.34 | >0.9 | 1.00 | 0.74, 1.36 | >0.9 | 1.02 | 0.75, 1.38 | 0.90 |
| Q3 | 1.10 | 0.81, 1.50 | 0.50 | 1.07 | 0.78, 1.46 | 0.70 | 1.09 | 0.79, 1.49 | 0.60 |
| Q4 | 1.07 | 0.83, 1.36 | 0.60 | 1.02 | 0.77, 1.34 | 0.90 | 1.07 | 0.81, 1.42 | 0.60 |
| *P*_trend_ | 0.50 | | | 0.80 | | | 0.60 | | |
| **2HPMA** | —— | —— | —— | —— | —— | —— | —— | —— | —— |
| Q2 | 1.03 | 0.81, 1.32 | 0.80 | 0.98 | 0.75, 1.28 | 0.90 | 0.98 | 0.74, 1.28 | 0.90 |
| Q3 | 0.97 | 0.72, 1.32 | 0.90 | 0.95 | 0.70, 1.30 | 0.80 | 0.98 | 0.72, 1.34 | >0.90 |
| Q4 | 0.97 | 0.73, 1.28 | 0.80 | 0.94 | 0.71, 1.25 | 0.70 | 0.95 | 0.71, 1.27 | 0.70 |
| *P*_trend_ | 0.80 | | | 0.70 | | | 0.80 | | |
| **SBMA** | —— | —— | —— | —— | —— | —— | —— | —— | —— |
| Q2 | 1.05 | 0.78, 1.41 | 0.70 | 0.98 | 0.71, 1.35 | 0.90 | 1.00 | 0.73, 1.38 | >0.90 |
| Q3 | 1.08 | 0.83, 1.39 | 0.60 | 0.92 | 0.70, 1.20 | 0.50 | 0.94 | 0.73, 1.22 | 0.60 |
| Q4 | 1.05 | 0.80, 1.38 | 0.70 | 0.88 | 0.66, 1.18 | 0.40 | 0.92 | 0.68, 1.24 | 0.60 |
| *P*_trend_ | 0.70 | | | 0.30 | | | 0.50 | | |
| **2MHA** | —— | —— | —— | —— | —— | —— | —— | —— | —— |
| Q2 | 0.79 | 0.59, 1.07 | 0.12 | 0.77 | 0.57, 1.05 | 0.10 | 0.78 | 0.58, 1.06 | 0.11 |
| Q3 | 0.75 | 0.54, 1.03 | 0.07 | 0.73 | 0.52, 1.04 | 0.08 | 0.73 | 0.52, 1.03 | 0.07 |
| Q4 | 0.58 | 0.45, 0.75 | **<0.01** | 0.52 | 0.39, 0.70 | **<0.01** | 0.53 | 0.40, 0.72 | **<0.01** |
| *P*_trend_ | **<0.01** | | | **<0.01** | | | **<0.01** | | |
| **3,4MHA** | —— | —— | —— | —— | —— | —— | —— | —— | —— |
| Q2 | 1.53 | 1.14, 2.05 | **<0.05** | 1.41 | 1.05, 1.88 | **<0.05** | 1.43 | 1.07, 1.89 | **<0.05** |
| Q3 | 1.12 | 0.83, 1.51 | 0.50 | 0.98 | 0.72, 1.33 | 0.90 | 0.99 | 0.73, 1.33 | >0.9 |
| Q4 | 0.89 | 0.65, 1.22 | 0.50 | 0.75 | 0.54, 1.05 | 0.10 | 0.77 | 0.54, 1.09 | 0.13 |
| *P*_trend_ | 0.20 | | | **<0.05** | | | **<0.05** | | |

***Notes:*** Urinary mVOCs were introduced into logistic regression models as categorical variables. All results were presented as OR, 95% *CI*, *P* value, and *P*_trend_. **Model1:** crude model with no covariate adjusted; **Model2:** adjusted for age, gender, race or ethnicity, education level, marriage status and poverty-income ratio; **Model3:** on the basis of model2, further adjusted for smoking status, alcohol drinking, daily total energy intake, physical activity, history of cancer.

***Abbreviations:*** mVOCs, metabolites of volatile organic compounds; FBG, fasting blood glucose; *OR*, odd ratio; 95% CI, 95% confident interval; CEMA, N-Acetyl-S-(2-carboxyethyl)-L-cysteine; 3HPMA, N-Acetyl-S-(3-hydroxypropyl)-L-cysteine; AAMA, N-Acetyl-S-(2-carbamoylethyl)-L-cysteine; CYMA, N-Acetyl-S-(2-cyanoethyl)-L-cysteine; DHBMA, N-Acetyl-S-(3,4-dihydroxybutyl)-L-cysteine; MHBMA3, N-Acetyl-S-(4-hydroxy-2-butenyl)-L-cysteine; HMPMA, N-Acetyl-S-(3-hydroxypropyl-1-methyl)-L-cysteine; ATCA, 2-Aminothiazoline-4-carboxylic acid; AMCC, N-Acetyl-S-(N-methylcarbamoyl)-L-cysteine; PGA, Phenylglyoxylic acid; MA, Mandelic acid; 2HPMA, N-Acetyl-S-(2-hydroxypropyl)-L-cysteine; SBMA, N-Acetyl-S-(benzyl)-L-cysteine; 2MHA, 2-Methylhippuric acid; 3,4-MHA, 3- and 4-Methylhippuric acid.

**Table.S7** Association between urine mVOCs and MetS stratified by gender subgroups

| **Urinary mVOCs** | **Male** | | | **Female** | | |
| --- | --- | --- | --- | --- | --- | --- |
|  | ***OR*** | **95% *CI*** | **P value** | ***OR*** | **95% *CI*** | **P value** |
| **CEMA** | —— | —— | —— | —— | —— | —— |
| Q2 | 1.68 | 1.18, 2.40 | **<0.05** | 1.19 | 0.87, 1.63 | 0.30 |
| Q3 | 1.51 | 1.01, 2.28 | **<0.05** | 1.69 | 1.21, 2.36 | **<0.01** |
| Q4 | 1.91 | 1.26, 2.90 | **<0.01** | 1.72 | 1.26, 2.36 | **<0.01** |
| *P*_trend_ | **<0.05** |  |  | **<0.01** |  |  |
| **3HPMA** | —— | —— | —— | —— | —— | —— |
| Q2 | 1.17 | 0.77, 1.77 | 0.50 | 1.36 | 0.84, 2.20 | 0.20 |
| Q3 | 1.85 | 1.24, 2.76 | **<0.01** | 1.28 | 0.89, 1.85 | 0.20 |
| Q4 | 1.37 | 0.87, 2.17 | 0.20 | 1.27 | 0.82, 1.97 | 0.30 |
| *P*_trend_ | 0.07 |  |  | 0.30 |  |  |
| **AAMA** | —— | —— | —— | —— | —— | —— |
| Q2 | 1.06 | 0.77, 1.46 | 0.70 | 0.62 | 0.42, 0.93 | **<0.05** |
| Q3 | 1.08 | 0.74, 1.56 | 0.70 | 0.77 | 0.54, 1.09 | 0.14 |
| Q4 | 0.73 | 0.49, 1.09 | 0.12 | 0.72 | 0.53, 0.99 | **<0.05** |
| *P*_trend_ | 0.20 |  |  | 0.12 |  |  |
| **CYMA** | —— | —— | —— | —— | —— | —— |
| Q2 | 1.03 | 0.70, 1.52 | 0.90 | 0.83 | 0.58, 1.20 | 0.30 |
| Q3 | 0.79 | 0.57, 1.10 | 0.20 | 0.97 | 0.69, 1.37 | 0.90 |
| Q4 | 0.83 | 0.52, 1.32 | 0.40 | 0.97 | 0.62, 1.50 | 0.90 |
| *P*_trend_ | 0.20 |  |  | >0.90 |  |  |
| **DHBMA** | —— | —— | —— | —— | —— | —— |
| Q2 | 1.07 | 0.74, 1.55 | 0.70 | 1.05 | 0.73, 1.51 | 0.80 |
| Q3 | 1.23 | 0.82, 1.84 | 0.30 | 1.01 | 0.63, 1.62 | >0.9 |
| Q4 | 1.51 | 0.93, 2.45 | 0.09 | 1.43 | 0.94, 2.18 | 0.10 |
| *P*_trend_ | 0.08 |  |  | 0.20 |  |  |
| **MHBMA3** | —— | —— | —— | —— | —— | —— |
| Q2 | 1.25 | 0.89, 1.75 | 0.20 | 1.36 | 0.99, 1.88 | 0.06 |
| Q3 | 1.41 | 1.02, 1.97 | **<0.05** | 1.40 | 0.95, 2.05 | 0.08 |
| Q4 | 1.14 | 0.71, 1.83 | 0.60 | 1.34 | 0.99, 1.81 | 0.06 |
| *P*_trend_ | 0.50 |  |  | 0.07 |  |  |
| **HMPMA** | —— | —— | —— | —— | —— | —— |
| Q2 | 1.61 | 1.13, 2.28 | **<0.05** | 1.14 | 0.75, 1.72 | 0.50 |
| Q3 | 1.95 | 1.31, 2.91 | **<0.01** | 1.86 | 1.23, 2.79 | **<0.01** |
| Q4 | 1.52 | 1.03, 2.24 | **<0.05** | 1.69 | 1.11, 2.57 | **<0.05** |
| *P*_trend_ | **<0.05** |  |  | **<0.01** |  |  |
| **ATCA** | —— | —— | —— | —— | —— | —— |
| Q2 | 0.99 | 0.76, 1.29 | >0.90 | 0.60 | 0.39, 0.93 | **<0.05** |
| Q3 | 1.05 | 0.77, 1.45 | 0.70 | 0.66 | 0.42, 1.02 | 0.06 |
| Q4 | 1.38 | 0.87, 2.21 | 0.20 | 0.63 | 0.41, 0.97 | **<0.05** |
| *P*_trend_ | 0.20 |  |  | 0.06 |  |  |
| **AMCC** | —— | —— | —— | —— | —— | —— |
| Q2 | 0.80 | 0.56, 1.14 | 0.20 | 0.78 | 0.52, 1.17 | 0.20 |
| Q3 | 0.71 | 0.47, 1.05 | 0.09 | 0.72 | 0.50, 1.03 | 0.07 |
| Q4 | 0.74 | 0.49, 1.11 | 0.14 | 0.54 | 0.34, 0.84 | **<0.05** |
| *P*_trend_ | 0.13 |  |  | **<0.05** |  |  |
| **PGA** | —— | —— | —— | —— | —— | —— |
| Q2 | 0.87 | 0.61, 1.26 | 0.50 | 0.67 | 0.47, 0.96 | **<0.05** |
| Q3 | 0.89 | 0.60, 1.31 | 0.50 | 0.64 | 0.43, 0.96 | **<0.05** |
| Q4 | 0.70 | 0.47, 1.03 | 0.07 | 0.59 | 0.40, 0.87 | **<0.05** |
| *P*_trend_ | 0.08 |  |  | **<0.05** |  |  |
| **MA** | —— | —— | —— | —— | —— | —— |
| Q2 | 0.78 | 0.51, 1.18 | 0.20 | 1.04 | 0.70, 1.53 | 0.90 |
| Q3 | 0.92 | 0.57, 1.48 | 0.70 | 1.07 | 0.72, 1.59 | 0.70 |
| Q4 | 0.79 | 0.59, 1.08 | 0.14 | 1.17 | 0.77, 1.76 | 0.50 |
| *P*_trend_ | 0.40 |  |  | 0.50 |  |  |
| **2HPMA** | —— | —— | —— | —— | —— | —— |
| Q2 | 1.15 | 0.80, 1.65 | 0.50 | 1.25 | 0.88, 1.77 | 0.20 |
| Q3 | 1.13 | 0.74, 1.74 | 0.60 | 0.82 | 0.57, 1.16 | 0.30 |
| Q4 | 1.07 | 0.71, 1.61 | 0.70 | 0.75 | 0.51, 1.11 | 0.20 |
| *P*_trend_ | 0.80 |  |  | **<0.05** |  |  |
| **SBMA** | —— | —— | —— | —— | —— | —— |
| Q2 | 1.01 | 0.69, 1.46 | >0.90 | 0.81 | 0.53, 1.22 | 0.30 |
| Q3 | 1.10 | 0.77, 1.55 | 0.60 | 0.83 | 0.56, 1.21 | 0.30 |
| Q4 | 0.87 | 0.60, 1.27 | 0.50 | 0.74 | 0.51, 1.08 | 0.12 |
| *P*_trend_ | 0.50 |  |  | 0.14 |  |  |
| **2MHA** | —— | —— | —— | —— | —— | —— |
| Q2 | 0.80 | 0.57, 1.13 | 0.20 | 0.90 | 0.64, 1.26 | 0.50 |
| Q3 | 0.99 | 0.68, 1.44 | >0.90 | 0.68 | 0.47, 1.01 | 0.05 |
| Q4 | 0.79 | 0.53, 1.18 | 0.20 | 0.47 | 0.30, 0.74 | **<0.01** |
| *P*_trend_ | 0.40 |  |  | **<0.01** |  |  |
| **3,4MHA** | —— | —— | —— | —— | —— | —— |
| Q2 | 1.15 | 0.79, 1.66 | 0.50 | 1.12 | 0.75, 1.68 | 0.60 |
| Q3 | 1.29 | 0.97, 1.71 | 0.08 | 0.76 | 0.49, 1.19 | 0.20 |
| Q4 | 0.86 | 0.54, 1.38 | 0.50 | 0.61 | 0.38, 0.97 | **<0.05** |
| *P*_trend_ | 0.60 |  |  | **<0.05** |  |  |

***Notes:*** Urinary mVOCs were introduced into logistic regression models as categorical variables. All results were presented as OR, 95% *CI*, *P* value, and *P*_trend_. Models were all adjusted for age, race or ethnicity, education level, marriage status, poverty-income ratio, smoking status, alcohol drinking, daily total energy intake, physical activity, and history of cancer.

***Abbreviations*:** mVOCs, metabolites of volatile organic compounds; MetS, metabolic syndrome; *OR*, odd ratio; 95% CI, 95% confident interval; CEMA, N-Acetyl-S-(2-carboxyethyl)-L-cysteine; 3HPMA, N-Acetyl-S-(3-hydroxypropyl)-L-cysteine; AAMA, N-Acetyl-S-(2-carbamoylethyl)-L-cysteine; CYMA, N-Acetyl-S-(2-cyanoethyl)-L-cysteine; DHBMA, N-Acetyl-S-(3,4-dihydroxybutyl)-L-cysteine; MHBMA3, N-Acetyl-S-(4-hydroxy-2-butenyl)-L-cysteine; HMPMA, N-Acetyl-S-(3-hydroxypropyl-1-methyl)-L-cysteine; ATCA, 2-Aminothiazoline-4-carboxylic acid; AMCC, N-Acetyl-S-(N-methylcarbamoyl)-L-cysteine; PGA, Phenylglyoxylic acid; MA, Mandelic acid; 2HPMA, N-Acetyl-S-(2-hydroxypropyl)-L-cysteine; SBMA, N-Acetyl-S-(benzyl)-L-cysteine; 2MHA, 2-Methylhippuric acid; 3,4-MHA, 3- and 4-Methylhippuric acid.

**Table.S8** Association between urine mVOCs and MetS stratified by age subgroups

| **Urinary mVOCs** | Aged 20-59 years | | | Aged 60 and above years | | |
| --- | --- | --- | --- | --- | --- | --- |
|  | ***OR*** | 95% ***CI*** | ***P*** value | ***OR*** | 95% ***CI*** | *P* value |
| **CEMA** | —— | —— | —— | —— | —— | —— |
| Q2 | 1.46 | 1.11, 1.93 | **<0.05** | 1.44 | 0.90, 2.30 | 0.12 |
| Q3 | 1.68 | 1.25, 2.27 | **<0.01** | 1.46 | 0.90, 2.36 | 0.12 |
| Q4 | 1.98 | 1.37, 2.85 | **<0.01** | 1.58 | 1.01, 2.48 | **<0.05** |
| *P*_trend_ | **<0.01** | | | 0.06 | | |
| **3HPMA** | —— | —— | —— | —— | —— | —— |
| Q2 | 1.26 | 0.82, 1.91 | 0.30 | 1.30 | 0.85, 1.97 | 0.20 |
| Q3 | 1.28 | 0.91, 1.79 | 0.20 | 2.13 | 1.41, 3.20 | **<0.01** |
| Q4 | 1.24 | 0.77, 1.97 | 0.40 | 1.42 | 0.91, 2.23 | 0.12 |
| *P*_trend_ | 0.40 |  |  | 0.04 |  |  |
| **AAMA** | —— | —— | —— | —— | —— | —— |
| Q2 | 1.01 | 0.79, 1.29 | >0.90 | 0.62 | 0.41, 0.92 | 0.02 |
| Q3 | 1.12 | 0.82, 1.52 | 0.50 | 0.59 | 0.44, 0.78 | **<0.01** |
| Q4 | 0.75 | 0.51, 1.09 | 0.13 | 0.67 | 0.43, 1.06 | 0.09 |
| *P*_trend_ | 0.20 |  |  | 0.07 |  |  |
| **CYMA** | —— | —— | —— | —— | —— | —— |
| Q2 | 0.89 | 0.62, 1.28 | 0.50 | 1.04 | 0.70, 1.55 | 0.80 |
| Q3 | 0.74 | 0.54, 1.01 | 0.05 | 1.21 | 0.80, 1.85 | 0.40 |
| Q4 | 0.72 | 0.48, 1.07 | 0.10 | 1.01 | 0.59, 1.72 | >0.9 |
| *P*_trend_ | 0.05 |  |  | 0.80 |  |  |
| **DHBMA** | —— | —— | —— | —— | —— | —— |
| Q2 | 1.08 | 0.83, 1.41 | 0.60 | 1.05 | 0.60, 1.82 | 0.90 |
| Q3 | 1.23 | 0.88, 1.70 | 0.20 | 0.95 | 0.58, 1.55 | 0.80 |
| Q4 | 1.68 | 1.17, 2.42 | **<0.05** | 1.33 | 0.81, 2.20 | 0.30 |
| *P*_trend_ | **<0.05** |  |  | 0.30 |  |  |
| **MHBMA3** | —— | —— | —— | —— | —— | —— |
| Q2 | 1.32 | 1.03, 1.68 | 0.03 | 1.33 | 0.82, 2.14 | 0.20 |
| Q3 | 1.26 | 0.94, 1.69 | 0.12 | 1.64 | 1.01, 2.68 | 0.05 |
| Q4 | 1.15 | 0.83, 1.60 | 0.40 | 1.41 | 0.81, 2.46 | 0.20 |
| *P*_trend_ | 0.50 |  |  | 0.20 |  | = |
| **HMPMA** | —— | —— | —— | —— | —— | —— |
| Q2 | 1.60 | 1.14, 2.26 | **<0.05** | 0.98 | 0.59, 1.63 | >0.9 |
| Q3 | 1.93 | 1.36, 2.73 | **<0.01** | 1.96 | 1.19, 3.23 | **<0.05** |
| Q4 | 1.51 | 1.06, 2.15 | **<0.05** | 1.82 | 1.06, 3.12 | **<0.05** |
| *P*_trend_ | **<0.05** |  |  | **<0.05** |  |  |
| **ATCA** | —— | —— | —— | —— | —— | —— |
| Q2 | 0.87 | 0.64, 1.18 | 0.40 | 1.00 | 0.67, 1.51 | >0.9 |
| Q3 | 0.91 | 0.67, 1.24 | 0.50 | 1.18 | 0.73, 1.90 | 0.50 |
| Q4 | 1.12 | 0.83, 1.52 | 0.40 | 0.91 | 0.57, 1.44 | 0.70 |
| *P*_trend_ | 0.40 |  |  | 0.90 |  |  |
| **AMCC** | —— | —— | —— | —— | —— | —— |
| Q2 | 0.86 | 0.64, 1.15 | 0.30 | 0.91 | 0.56, 1.49 | 0.70 |
| Q3 | 0.72 | 0.48, 1.08 | 0.11 | 0.93 | 0.59, 1.46 | 0.70 |
| Q4 | 0.66 | 0.43, 1.03 | 0.07 | 0.82 | 0.49, 1.37 | 0.40 |
| *P*_trend_ | 0.07 |  |  | 0.50 |  |  |
| **PGA** | —— | —— | —— | —— | —— | —— |
| Q2 | 0.80 | 0.61, 1.06 | 0.12 | 0.73 | 0.47, 1.14 | 0.20 |
| Q3 | 0.84 | 0.63, 1.13 | 0.30 | 0.73 | 0.49, 1.07 | 0.10 |
| Q4 | 0.76 | 0.51, 1.14 | 0.20 | 0.57 | 0.36, 0.92 | **<0.05** |
| *P*_trend_ | 0.20 |  |  | **<0.05** |  |  |
| **MA** | —— | —— | —— | —— | —— | —— |
| Q2 | 0.88 | 0.61, 1.27 | 0.50 | 0.79 | 0.52, 1.20 | 0.30 |
| Q3 | 0.94 | 0.63, 1.41 | 0.80 | 0.93 | 0.61, 1.40 | 0.70 |
| Q4 | 0.93 | 0.66, 1.31 | 0.70 | 1.00 | 0.67, 1.49 | >0.90 |
| *P*_trend_ | 0.80 |  |  | 0.80 |  |  |
| **2HPMA** | —— | —— | —— | —— | —— | —— |
| Q2 | 1.34 | 0.97, 1.85 | 0.07 | 0.91 | 0.63, 1.32 | 0.60 |
| Q3 | 0.95 | 0.72, 1.27 | 0.70 | 0.96 | 0.66, 1.41 | 0.80 |
| Q4 | 0.77 | 0.54, 1.09 | 0.14 | 1.07 | 0.69, 1.66 | 0.80 |
| *P*_trend_ | 0.06 |  |  | 0.70 |  |  |
| **SBMA** | —— | —— | —— | —— | —— | —— |
| Q2 | 0.95 | 0.68, 1.33 | 0.80 | 0.87 | 0.52, 1.45 | 0.60 |
| Q3 | 0.93 | 0.69, 1.24 | 0.60 | 1.05 | 0.66, 1.66 | 0.80 |
| Q4 | 0.88 | 0.67, 1.16 | 0.40 | 0.85 | 0.51, 1.42 | 0.50 |
| *P*_trend_ | 0.30 |  |  | 0.70 |  |  |
| **2MHA** | —— | —— | —— | —— | —— | —— |
| Q2 | 0.75 | 0.55, 1.00 | 0.05 | 1.16 | 0.77, 1.76 | 0.50 |
| Q3 | 0.79 | 0.55, 1.13 | 0.20 | 0.92 | 0.57, 1.47 | 0.70 |
| Q4 | 0.64 | 0.40, 1.00 | 0.05 | 0.60 | 0.39, 0.92 | **<0.05** |
| *P*_trend_ | 0.08 |  |  | **<0.05** |  |  |
| **3,4MHA** | —— | —— | —— | —— | —— | —— |
| Q2 | 1.04 | 0.73, 1.48 | 0.80 | 1.49 | 1.00, 2.22 | 0.05 |
| Q3 | 0.97 | 0.70, 1.35 | 0.90 | 1.12 | 0.68, 1.83 | 0.70 |
| Q4 | 0.73 | 0.45, 1.18 | 0.20 | 0.76 | 0.42, 1.38 | 0.40 |
| *P*_trend_ | 0.20 |  |  | 0.20 |  |  |

***Notes:*** Urinary mVOCs were introduced into logistic regression models as categorical variables. All results were presented as OR, 95% *CI*, *P* value, and *P*_trend_. Models were all adjusted for gender, race or ethnicity, education level, marriage status, poverty-income ratio, smoking status, alcohol drinking, daily total energy intake, physical activity, and history of cancer.

***Abbreviations*:** mVOCs, metabolites of volatile organic compounds; MetS, metabolic syndrome; *OR*, odd ratio; 95% CI, 95% confident interval; CEMA, N-Acetyl-S-(2-carboxyethyl)-L-cysteine; 3HPMA, N-Acetyl-S-(3-hydroxypropyl)-L-cysteine; AAMA, N-Acetyl-S-(2-carbamoylethyl)-L-cysteine; CYMA, N-Acetyl-S-(2-cyanoethyl)-L-cysteine; DHBMA, N-Acetyl-S-(3,4-dihydroxybutyl)-L-cysteine; MHBMA3, N-Acetyl-S-(4-hydroxy-2-butenyl)-L-cysteine; HMPMA, N-Acetyl-S-(3-hydroxypropyl-1-methyl)-L-cysteine; ATCA, 2-Aminothiazoline-4-carboxylic acid; AMCC, N-Acetyl-S-(N-methylcarbamoyl)-L-cysteine; PGA, Phenylglyoxylic acid; MA, Mandelic acid; 2HPMA, N-Acetyl-S-(2-hydroxypropyl)-L-cysteine; SBMA, N-Acetyl-S-(benzyl)-L-cysteine; 2MHA, 2-Methylhippuric acid; 3,4-MHA, 3- and 4-Methylhippuric acid.

**Table.S9** Estimated weight of each LASSO-selected mVOC in WQS regression model

| **Outcomes** | **WQS model (Positive direction)** | | **WQS model (Negative direction)** | |
| --- | --- | --- | --- | --- |
|  | **Urinary mVOCs** | **Estimated weights** | **Urinary mVOCs** | **Estimated weights** |
| Central obesity | CEMA | 72.60% | 2HPMA | 34.00% |
|  | DHBMA | 25.70% | MA | 20.00% |
|  | SBMA | <0.01% | 2MHA | 19.00% |
|  | 3HPMA | <0.01% | SBMA | 15.00% |
|  | 2MHA | <0.01% | 3HPMA | 7.00% |
|  | MA | <0.01% | DHBMA | 3.00% |
|  | 2HPMA | <0.01% | CEMA | 3.00% |
| Elevated TG | 3HPMA | 12.50% | PGA | 12.00% |
|  | HMPMA | 12.00% | CYMA | 12.00% |
|  | CEMA | 11.60% | SBMA | 12.00% |
|  | MA | 11.00% | 2HPMA | 11.00% |
|  | AAMA | 10.70% | AAMA | 11.00% |
|  | 2HPMA | 10.60% | MA | 11.00% |
|  | CYMA | 10.50% | HMPMA | 11.00% |
|  | SBMA | 10.50% | CEMA | 11.00% |
|  | PGA | 10.50% | 3HPMA | 10.00% |
| Reduced HDL | CEMA | 17.50% | SBMA | 14.40% |
|  | HMPMA | 17.10% | PGA | 14.40% |
|  | MHBMA3 | 16.60% | AAMA | 14.30% |
|  | DHBMA | 13.60% | DHBMA | 14.30% |
|  | AAMA | 12.90% | CEMA | 14.20% |
|  | PGA | 11.40% | MHBMA3 | 14.20% |
|  | SBMA | 10.90% | HMPMA | 14.10% |
| High BP | CEMA | 66.40% | 2HPMA | 74.80% |
|  | 2HPMA | 33.60% | CEMA | 25.20% |
| Impaired FBG | HMPMA | 55.90% | 2MHA | 27.60% |
|  | CYMA | 16.50% | CYMA | 25.30% |
|  | AAMA | 16.10% | AAMA | 24.70% |
|  | 2MHA | 11.50% | HMPMA | 22.40% |

***Notes:*** The WQS models were adjusted for all covariates, including gender, age, race or ethnicity, education level, marriage status, poverty-income ratio, smoking status, alcohol drinking, daily total energy intake, physical activity, and history of cancer.

***Abbreviations*:** mVOCs, metabolites of volatile organic compounds; MetS, metabolic syndrome; TG, triglyceride; HDL, high-density lipoprotein; BP, blood pressure; FBG, fasting blood glucose; CEMA, N-Acetyl-S-(2-carboxyethyl)-L-cysteine; DHBMA, N-Acetyl-S-(3,4-dihydroxybutyl)-L-cysteine; 3HPMA, N-Acetyl-S-(3-hydroxypropyl)-L-cysteine; SBMA, N-Acetyl-S-(benzyl)-L-cysteine; 2MHA, 2-Methylhippuric acid; MA, Mandelic acid; 2HPMA, N-Acetyl-S-(2-hydroxypropyl)-L-cysteine; HMPMA, N-Acetyl-S-(3-hydroxypropyl-1-methyl)-L-cysteine; AAMA, N-Acetyl-S-(2-carbamoylethyl)-L-cysteine; CYMA, N-Acetyl-S-(2-cyanoethyl)-L-cysteine; PGA, Phenylglyoxylic acid; MHBMA3, N-Acetyl-S-(4-hydroxy-2-butenyl)-L-cysteine.


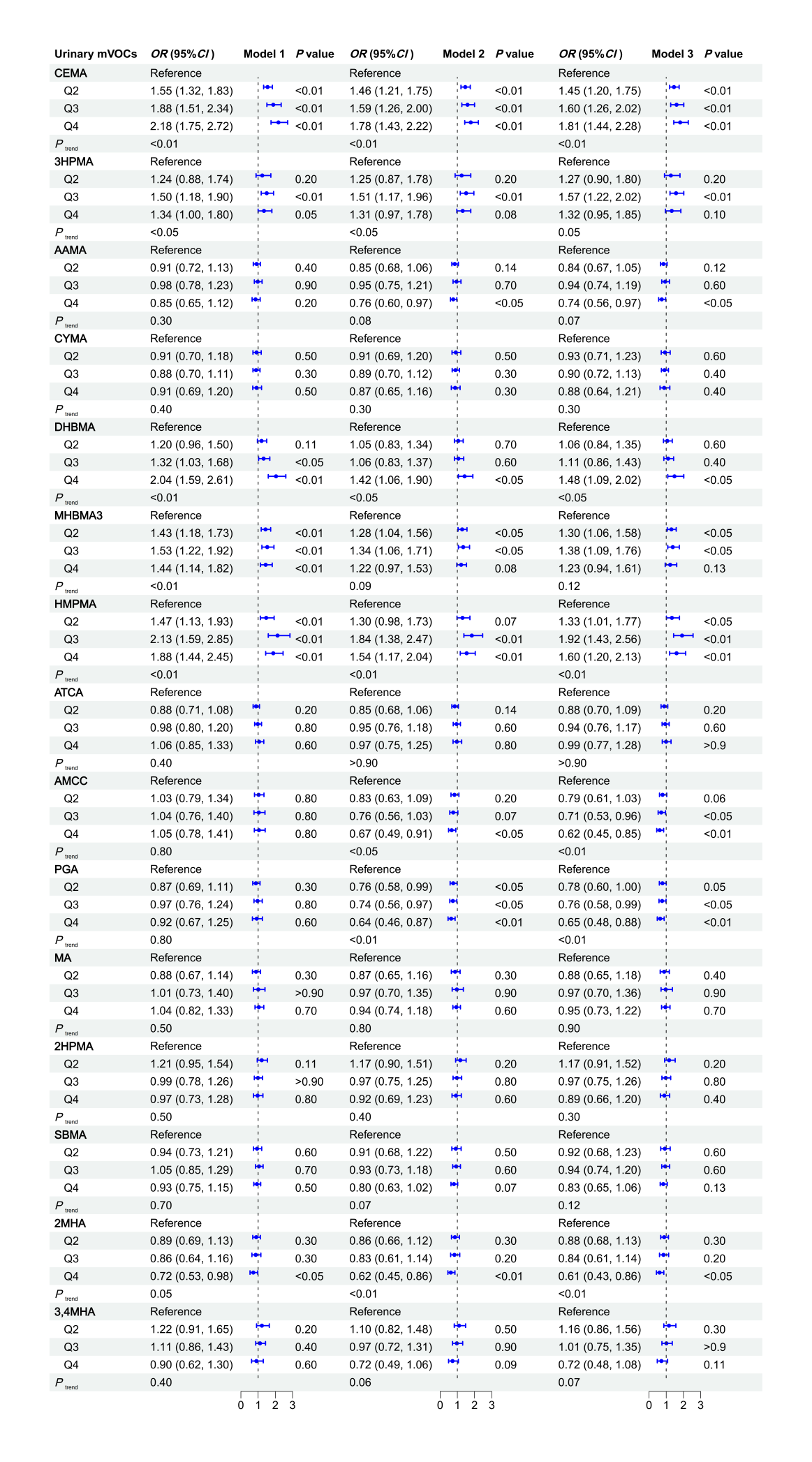


**Figure.S1** Forest plot of association between urinary mVOCs and MetS in multivariate logistic regression analysis

***Notes:*** Urinary mVOCs were introduced into logistic regression models as categorical variables. Model 1 was a crude model with no covariate adjusted; Model 2 was adjusted for age, gender, race or ethnicity, education level, marriage status and poverty-income ratio; Model 3 was further adjusted for smoking status, alcohol drinking, daily total energy intake, physical activity, and history of cancer based on the model2. All results were presented as OR, 95% CI, P value, and Ptrend.

***Abbreviations:*** mVOCs, metabolites of volatile organic compounds; MetS, metabolic syndrome; OR, odd ratio; 95% CI, 95% confident interval; CEMA, N-Acetyl-S-(2-carboxyethyl)-L-cysteine; 3HPMA, N-Acetyl-S-(3-hydroxypropyl)-L-cysteine; AAMA, N-Acetyl-S-(2-carbamoylethyl)-L-cysteine; CYMA, N-Acetyl-S-(2-cyanoethyl)-L-cysteine; DHBMA, N-Acetyl-S-(3,4-dihydroxybutyl)-L-cysteine; MHBMA3, N-Acetyl-S-(4-hydroxy-2-butenyl)-L-cysteine; HMPMA, N-Acetyl-S-(3-hydroxypropyl-1-methyl)-L-cysteine; ATCA, 2-Aminothiazoline-4-carboxylic acid; AMCC, N-Acetyl-S-(N-methylcarbamoyl)-L-cysteine; PGA, Phenylglyoxylic acid; MA, Mandelic acid; 2HPMA, N-Acetyl-S-(2-hydroxypropyl)-L-cysteine; SBMA, N-Acetyl-S-(benzyl)-L-cysteine; 2MHA, 2-Methylhippuric acid; 3,4-MHA, 3- and 4-Methylhippuric acid.
